# Expression profiling of rainbow trout testis development identifies evolutionary conserved genes involved in spermatogenesis

**DOI:** 10.1186/1471-2164-10-546

**Published:** 2009-11-20

**Authors:** Antoine D Rolland, Jean-Jacques Lareyre, Anne-Sophie Goupil, Jérôme Montfort, Marie-Jo Ricordel, Diane Esquerré, Karine Hugot, Rémi Houlgatte, Fréderic Chalmel, Florence Le Gac

**Affiliations:** 1 INRA, UR1037, IFR-140, Ouest Genopole, Rennes, 35042, France; 2 INRA, UMR 1313 de Génétique Animale et Biologie Intégrative, Domaine de Vilvert, 78350 Jouy-en-Josas, France; 3 CEA, DSV, iRCM, SREIT, Laboratoire de Génétique Animale et Biologie Intégrative, 78350 Jouy-en-Josas, France; 4 AgroParisTech, UMR de Génétique Animale et Biologie Intégrative, 78350 Jouy-en-Josas, France; 5 Inserm, U915, Université de Nantes, Faculté de Médecine, Nantes, 44035, France; 6 Inserm, U625, GERHM, Université de Rennes1, IFR-140, Ouest Genopole, Rennes, 35042, France

## Abstract

**Background:**

Spermatogenesis is a late developmental process that involves a coordinated expression program in germ cells and a permanent communication between the testicular somatic cells and the germ-line. Current knowledge regarding molecular factors driving male germ cell proliferation and differentiation in vertebrates is still limited and mainly based on existing data from rodents and human. Fish with a marked reproductive cycle and a germ cell development in synchronous cysts have proven to be choice models to study precise stages of the spermatogenetic development and the germ cell-somatic cell communication network. In this study we used 9K cDNA microarrays to investigate the expression profiles underlying testis maturation during the male reproductive cycle of the trout, *Oncorhynchus mykiss*.

**Results:**

Using total testis samples at various developmental stages and isolated spermatogonia, spermatocytes and spermatids, 3379 differentially expressed trout cDNAs were identified and their gene activation or repression patterns throughout the reproductive cycle were reported. We also performed a tissue-profiling analysis and highlighted many genes for which expression signals were restricted to the testes or gonads from both sexes. The search for orthologous genes in genome-sequenced fish species and the use of their mammalian orthologs allowed us to provide accurate annotations for trout cDNAs. The analysis of the GeneOntology terms therefore validated and broadened our interpretation of expression clusters by highlighting enriched functions that are consistent with known sequential events during male gametogenesis. Furthermore, we compared expression profiles of trout and mouse orthologs and identified a complement of genes for which expression during spermatogenesis was maintained throughout evolution.

**Conclusion:**

A comprehensive study of gene expression and associated functions during testis maturation and germ cell differentiation in the rainbow trout is presented. The study identifies new pathways involved during spermatogonia self-renewal or rapid proliferation, meiosis and gamete differentiation, in fish and potentially in all vertebrates. It also provides the necessary basis to further investigate the hormonal and molecular networks that trigger puberty and annual testicular recrudescence in seasonally breeding species.

## Background

The formation of male gametes, spermatozoa, occurs in the testes by means of a highly coordinated differentiation process called spermatogenesis that includes three phases. Firstly, primary germ cells or spermatogonia actively divide and after a fixed number of mitoses, differentiate to become spermatocytes. During meiosis, spermatocytes undergo DNA recombinations and then two successive divisions to produce haploid spermatids. Finally, during spermiogenesis, spermatids differentiate into mature, motile spermatozoa. In addition to a complex germ cell intrinsic genetic program, the entire spermatogenetic process also strongly relies on numerous endocrine and paracrine signalling pathways from testicular somatic cells [1-5]. In mammals, this cellular communication network that triggers efficient spermatogenesis notably involves the production of androgens by Leydig cells in response to the luteinizing hormone (LH) and the sustain of germ cell survival and differentiation by the so-called "nurse" Sertoli cells, which are themselves responsive to both androgens and the follicle-stimulating hormone (FSH). Whereas these overall features of spermatogenesis appear to have been conserved in vertebrates, a number of reproductive strategies have emerged throughout evolution, leading to marked differences in terms of structural organisation of the testes or regarding the frequency, timing and rate of spermatogenesis [6]. These spatio-temporal differences in the development of the germinal compartment are believed to involve original regulatory events.

In seasonally breeding rainbow trout, *Oncorhynchus mykiss*, spermatogenesis occurs during a marked annual reproductive cycle during which the testis undergoes a dramatic increase in size (from 0.05% up to 8% of the body weight), while germ cells develop partially synchronously, to finally allow the massive production of sperm within a short period of time once a year [7,8]. Many efforts have been made in commercial fish farms to control reproductive cycles in order to obtain all-year-round spawning fish or to prevent the slowing of growth linked to sexual maturation. Successful results have been obtained to some extent through manipulating the photoperiod or the hormonal status of fish. However, the mechanisms underlying such effects are still poorly understood and these applications in broodstock management remain limited to a few situations [9,10]. Further research efforts concerning the cellular pathways involved during spermatogenesis recrudescence are thus necessary if we are to understand and control testis maturation in teleost fish species. Moreover, the seasonally-driven testis development in trout provides a new model for deciphering the hormonal and paracrine regulatory processes that govern early germ cell proliferation and differentiation until spermatozoa secretion.

A microarray experiment investigating testicular gene expression during the reproductive cycle in trout is reported here. The use of entire gonad samples at various stages together with isolated germ cells allowed us to follow expression patterns during testis development and more importantly, to predict cellular origins of differentially-expressed genes. We also carried out a cross-species comparison and identified a complement of evolutionary conserved genes displaying very similar expression during spermatogenesis in trout and mice. Finally, by using non-testicular trout tissue expression data we found potential testis-specific genes that may have unique functions during spermatogenesis. Together with the confident annotations we provide for the genes represented on the trout microarray, this dataset thus constitutes a necessary and solid basis to further investigate the control of spermatogenesis in fish.

## Results

### Experimental design and overall microarray results

**Figure 1:**
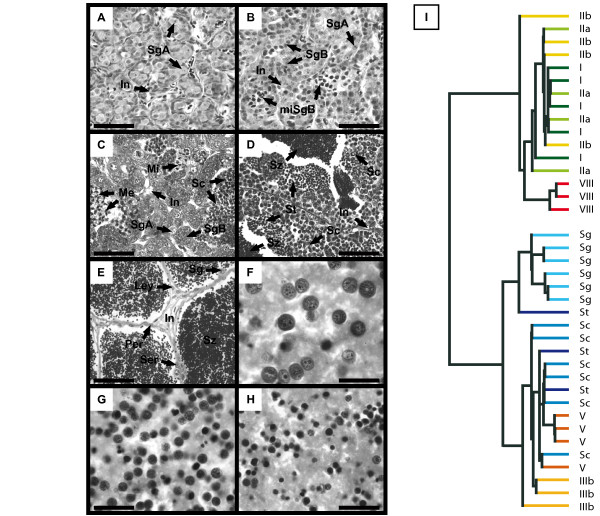
**Histological analysis of samples and classification of microarrays**. A-F. Histology of selected samples used in the microarray experiment. Characteristic developmental stages I (panel A), II (panel B), III (panel C), V (panel D) and VIII (panel E) are presented. Representative enriched fractions of isolated spermatogonia (F), spermatocytes (G) and spermatids (H) are also shown. Scale bars in panels A-E and F-H represent 50 and 20 μm, respectively. I. Dendogram of sample correlation according to hierarchical classification (uncentered correlation, average linkage) performed on entire microarray signals. Roman numerals (I-VIII) indicate trout developmental stages. Sg = Spermatogonia; SgA and SgB = Type-A and type-B spermatogonia, respectively; Sc = Spermatocytes; St = Spermatids; Sz = Spermatozoa; Ser = Sertoli cell; Ley = Leydig cell; Per = Peritubular cell; Me = Meiosis; Mi = Mitosis; In = Interstitial tissue.

Trout spermatogenesis occurs seasonally in such a way that all morphological and cellular events tend to be synchronized [11,12]. We thus used gonads at key stages in the male reproductive cycle (Figure 1A-E) to study the changes in gene expression underlying testis development. These included gonads at early stages containing slowly-dividing type A spermatogonia (Stage I, Figure 1A) or both type A and actively-dividing type B spermatogonia (Stages IIa and IIb, Figure 1B), maturing testes containing meiotic spermatocytes (Stage IIIb, Figure 1C) and post-meiotic spermatids (Stage V, Figure 1D) in addition to growing numbers of spermatogonia and, finally, gonads at a later stage, i.e. spawning testes, containing essentially fully developed spermatozoa (stage VIII, Figure 1E). Additionally, fractions of isolated germ cells enriched in spermatogonia (Figure 1F), spermatocytes (Figure 1G) or spermatids (Figure 1H) were used to identify the cellular origins of measured expression signals.

Unsupervised hierarchical classification of the samples (Figure 1I) clearly distinguished immature (Stages I, IIa and IIb) and spawning (Stages VIII) gonads from isolated germ cell fractions and maturing gonads undergoing meiosis and spermiogenesis (Stages IIIb and V, respectively). The correlation observed between isolated germ cell samples and total testes harbouring active spermatogenesis is not surprising given the large number of differentiating germ cells that accumulate during testis maturation. Inversely, the correlation observed between the early stages and stage VIII is more intriguing: Gonads in the early stages contain mainly spermatogonia under various proliferation states together with somatic cells (i.e. Leydig, Sertoli and peritubular myoid cells), whereas stage VIII gonads are predominantly composed of mature spermatozoa and somatic cells (Figure 1H). Since spermatozoa only contain very small amounts of RNA [13], expression signals from stage VIII gonads are actually likely to represent the somatic complement of spawning testes. The overall expression profiles from entire gonads thus appeared consistent with the cellular events taking place throughout the male trout reproductive cycle.

### Annotation of the trout cDNA microarray

Genes/transcripts corresponding to the EST sequences available for the 9024 clones spotted on the microarray were annotated by searching for potential orthologous genes in the genome of model fish species - for instance Gasterosteus aculeatus, Danio rerio, Oryzias latipes and Takifugu rubripes. Among the 8665 clones mapped on at least one of these fish genomes (BLAT algorithm), 8647 were associated with Ensembl gene IDs. Overall, 8197 trout clones were thus confidently linked to a model fish gene - i.e. with at least 2 EST hits across all 4 genomes leading to the same Ensembl gene or to similar genes encoding proteins belonging to the same family - and corresponded to 6661 non redundant (NR) genes. We next used information from the Ensembl database to associate clones with GeneOntology (GO) terms [14]. To increase the somehow weak annotation of fish genes, GO terms associated with corresponding mammalian genes were also used. This inference was possible for 7065 clones for which associated fish genes also had rat, mouse and/or human orthologous genes, as predicted by the Ensembl database (Compara, version 52). This strategy enabled us to annotate 88% of the spotted clones in terms of "biological process", "molecular function" or "cellular component" annotations.

### Identification of differentially-expressed genes during trout testis spermatogenesis

**Figure 2:**
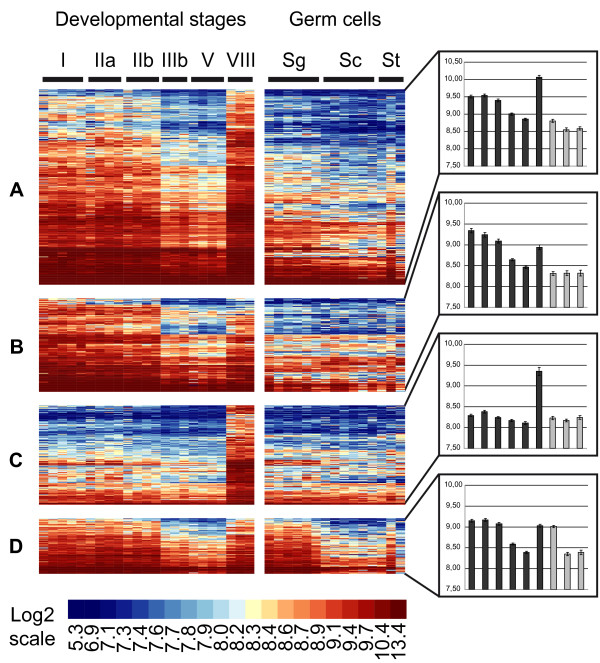
**Somatic expression clusters during rainbow trout spermatogenesis**. Heatmap representation of genes preferentially expressed in somatic cells during trout spermatogenesis. After statistical filtrations, co-expressed genes were classified in 9 clusters (A-I, see also figure 3) according to expression profiles in developmental stages and isolated germ cells using the PAM algorithm. Each line represents the expression signal of a single clone and each column is a sample. Roman numerals (I-VIII) indicate testicular developmental stages. Sg = spermatogonia; Sc = spermatocytes; St = spermatids. Log-2 transformed signal intensities are shown according to the scale bar. Median expression profiles for each cluster are also presented (Histograms, right panels).

**Figure 3:**
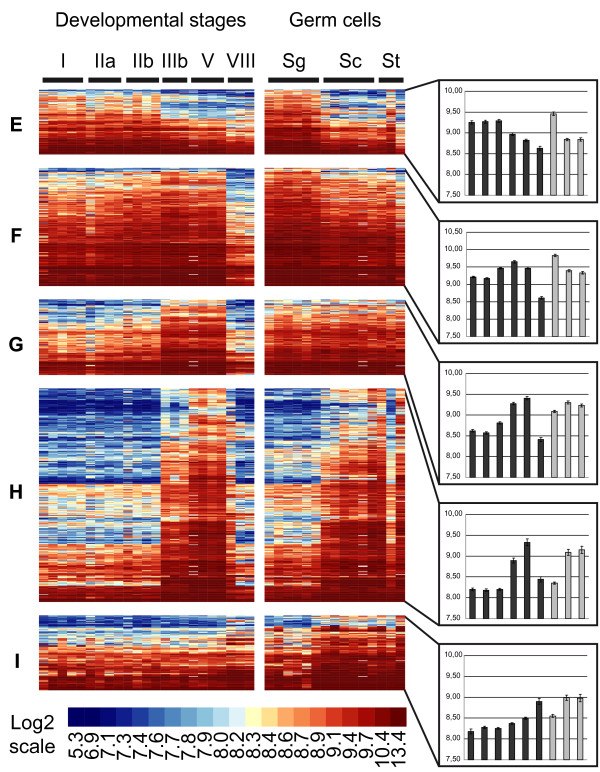
**Germ cell expression clusters during rainbow trout spermatogenesis**. Heatmap representation of genes preferentially expressed in germ cells during trout spermatogenesis. After statistical filtrations, co-expressed genes were classified in 9 clusters (A-I, see also figure 2) according to expression profiles in developmental stages and isolated germ cells using the PAM algorithm. Each line represents the expression signal of a single clone and each column is a sample. Roman numerals (I-VIII) indicate testicular developmental stages. Sg = spermatogonia; Sc = spermatocytes; St = spermatids. Log-2 transformed signal intensities are shown according to the scale bar. Median expression profiles for each cluster are also presented (Histograms, right panels).

A statistical analysis, based on the comparison of 38 samples hybridized onto 38 microarrays, was performed to identify genes exhibiting significant changes in expression during trout spermatogenesis. Among 7821 well-measured clones, 3379 were thus found to be differentially-expressed and corresponded to 2771 NR genes (Additional file 1). Their classification in 9 expression clusters then allowed the identification of sequential events of gene activation or repression during testis development and/or germ cell differentiation. To further distinguish somatic cell from germ cell transcripts we focused on 1694 highly differentially-expressed clones (1441 NR genes; labelled "High differential" in additional file 1) for which cellular origins can be more confidently inferred (Figures 2 and 3). Note that the color-code used for heatmap representations reflects the signal intensity, i.e. the expression level, of a given gene, ranging from not detected (dark blue) to highly expressed (red). It therefore allows not only to represent changes in expression for a given gene, but also to compare expression levels between genes.

At least 3 clusters corresponding to genes preferentially-expressed in somatic cells, as evidenced by their low or null expression in isolated germ cell samples, could thus be distinguished. Genes in cluster A (334 clones/284 NR genes) are expressed in early developmental stages, then decrease by a dilution-effect as germ cells accumulate within the gonads (from stage IIb onwards) and finally increase in stage VIII testes when very few germ cells, apart from spermatozoa, are found within the testis (Figure 2). In contrast to this archetypical somatic expression pattern, genes in clusters B (160/133) and C (170/141) are expressed at lower and higher levels in stage VIII gonads, respectively (Figure 2). These genes are therefore likely to be subjected to differential expression regulations during the reproductive cycle in comparison with cluster A. Genes in cluster D (95/79) exhibit a somatic developmental expression pattern that is somehow similar to cluster A but are, moreover, highly expressed in isolated spermatogonia (Figure 2).

Clusters E and F contain genes that are most highly expressed in isolated spermatogonia but that could be distinguished on the basis of their developmental expression pattern (Figure 3). Genes in cluster E (111/94) exhibit a progressive decrease in expression as spermatogenesis proceeds (from stage IIIb onwards) and might correspond to weakly differentiated spermatogonia. In contrast, genes in cluster F (202/154) transiently increase from stages IIb to V and could correspond to more differentiated, actively-dividing spermatogonia and early spermatocytes that accumulate during the course of the reproductive cycle (Figures 3).

Clusters G, H and I were found to contain genes expressed within the germline or with expression peaking in meiotic and post-meiotic germ cells. Cluster G (129/108) indeed contains genes highly expressed in all germ cell types that are also highly expressed in stage IIIb and V gonads (Figure 3). Cluster H (365/302) corresponds to genes more specifically expressed in both spermatocytes and spermatids. Again, these transcripts are highly accumulating in stage IIIb and V gonads (Figure 3). Finally, genes belonging to cluster I (128/116) are highly expressed within the germline, especially in both spermatocytes and spermatids, but differ from clusters G and H as they do not show a clear differential expression pattern during testis maturation (Figure 3).

Normalized expression data, expression cluster information and corresponding annotations are available for all differentially-expressed trout clones in a searchable excel spreadsheet provided in additional file 1. Note that the expression profiles for 5 somatic genes (Amh, Dmrt1, Slc26a4, Sox9a and Tbx1) were verified and confirmed by qPCR experiments (Additional file 2). The comparison of the 2 methods revealed that microarrays data tend to minimize the differences between experimental groups of samples.

### Functional mining of expression clusters highlights relevant functions in trout testis development

**Figure 4:**
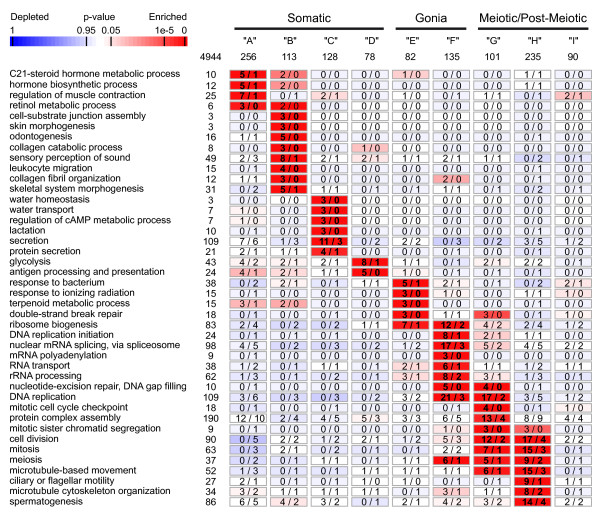
**Functional mining of trout spermatogenetic clusters**. Over-represented "biological process" terms from the GeneOntology (GO) were identified in the 9 expression clusters (A-I) previously identified. Rectangles indicate the observed (left) and expected (right) numbers of genes bearing the corresponding GO term, whereas the number of genes exhibiting this GO term on the entire microarray is given on the left. Only GO terms with a p-value ≤10^-6 ^and for which at least 3 non-redundant genes belonged to the cluster were considered as statistically-enriched. To avoid redundancy between closely related terms, an Ontology Specific Information Rate (OSIR) cut-off ≥ 0.95 was selected. Numbers in bold indicate a statistical enrichment for a given GO term according to the scale bar.

We performed a GeneOntology term analysis to investigate the biological significance of the 9 expression clusters described above. We found enriched functions in each cluster that are consistent with known events occurring at different stages of spermatogenesis and in specific cellular compartments of the testis ("biological process" terms, Figure 4).

The somatic expression cluster A was thus found to be significantly enriched in genes coding for proteins involved in hormone biosynthetic or catabolic processes, which may originate from steroidogenic Leydig cells, including some genes more specifically involved in C21-steroid hormone or retinol metabolic processes (Akr1d1, Cyp17a1, Cyp11a1, Dhrs3 and Dhrs9, Ece1, Hsd11b2). Additionally, a number of genes involved in the regulation of muscle contraction (Anxa6, Atp1a1, Gucy1a3, Myl9, Tpm1 and Tpm3) were found in this cluster and are possibly expressed by contractile peritubular myoid cells. Cluster B, which might correspond to somatic genes undergoing down regulation as the testis matures, was found to contain many genes involved in various developmental processes. It included genes encoding extracellular matrix constituents or organizers (Itga6, Col1a1 and Col1a2), but also transcription factors (Bapx1, Pcgf2, Sox10 and Tbx1) involved in skin and skeletal system morphogenesis or ondontogenesis. Genes in cluster C are highly expressed in stage VIII testes and were notably found to be involved in water transport and homeostasis, cAMP metabolic processes (Aqp4, Itnp, Timp2 and Vsnp), lactation and secretion (Ank1, Agpat9, Canx, Cav2, Dgat1, Gars, Glrx5 and Slc39a1). These biological processes appear to be highly relevant functions for semen fluid constitution and sperm hydration during spawning. Cluster D, which corresponds to genes that might be expressed in both somatic cells and spermatogonia, contains genes involved in glycolysis (Aldoa, Eno1p, Eno3, Gapdh, Pgk1 and Tpi1a), antigen processing and presentation (Cd74, B2m, Mhc1uca and Mica) and the aldehyde metabolic process (Tpia1, Aldh7a1 and Aldh9a1). Type A spermatogonia cluster E was found to be enriched in genes involved in the response to bacterium and ionizing radiation or terpenoid metabolic process (Adh5, C10orf33, Dhrs4, Gpx3, Hadha, Hamp1 and Tf). More interestingly, these genes involved in double strand break repair (Xrcc4 and Xrcc6 and Msh2) may participate in specific checkpoints in these cells to ensure the integrity of the transmitted genome. Additionally, numerous genes were involved in ribosome biogenesis (Aatf, Exosc5, Mina, Nmd3, Rplp0, Rpsa and Wdr3), a function that was also found to be enriched in cluster F. Cluster F is likely to correspond to more differentiated spermatogonia and was found to contain genes involved in RNA metabolism, including mRNA splicing, via spliceosome, RNA transport (Ckap5, Ddx39, Dye, Nup43, Thoc4 and Xpo1), rRNA processing (Ddx56, Dkc1, Eif4a3, Exocs7, Exocs8, Fbl, Nol5 and Utp14c) or mRNA polyadenylation (Cpsf1, Cstf2 and Pabpn1), which could be important for fine-tuning gene expression in these cells. In agreement with the high proliferative activity of these cells, a number of genes were also involved in DNA metabolic processes, notably in the initiation of DNA replication (Mcm2-7, Orcl1 and Rpa4) and nucleotide-excision repair, DNA gap filling or base excision repair (Msh6, Pcna, Pold1, Rfc3, Rfc4, Ung and Rpa4). Cluster G corresponds to genes widely expressed within the germline that are involved in DNA replication along with cell division, mitosis or mitotic cell cycle checkpoint (Cdc2, Mad2l1, Ttk and Zw10). Furthermore, genes involved in meiosis were found in cluster F (Fancd2, Kif2c, Msh6, Psmd13, Spin1, and Utp14c), cluster G (Psmc3ip, Rad50, Sycp3l, Ttk and Zw10) and cluster H as well (Ccna1, Ccnb3, Cdc25c, Dmc1, Kif2c, Meig1, Nek2, Topbp1 and Tsga2). Finally, whereas the meiotic/post-meiotic cluster H shared common biological functions with cluster G (Cell division, mitosis and microtubule-based movement), it was also consistently and specifically enriched in genes involved in microtubule cytoskeleton organization, ciliary or flagellar mobility (Cetn3, C14orf143, Dnah1, Dnah7, Dnah8, Dnah12, Fam179b, Gabarap, Kif2c, LOC795513, Ndel1, Tekt3, Tekt4, Tubb1 and Ube2c) and spermatogenesis (Ccna1, Dmc1, Dnah9, Dnajb13, Ggnbp2, Hist1h1d, Klhl10, Nme5, Ropn1l, Sapg6, Spata4, Tegt, Tle3 and Txndc6). Only cluster I did not show any specifically enriched biological processes and its biological significance still remains unclear.

Taken together, the statistical enrichments obtained in this GO term analysis validate our gene expression analysis and the annotation of trout genes. It can be noted that concordant results were obtained at molecular function or cellular component levels (Additional files 3 and 4) as well as for conserved genes correlated with mice and for testis-specific genes (see below and additional files 5 and 6).

### Correlating gene expression during spermatogenesis in fish and mammals

**Figure 5:**
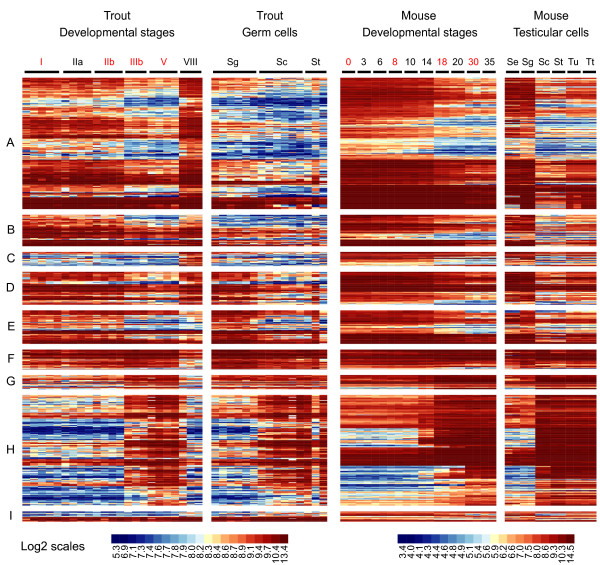
**Correlated expression of conserved genes during spermatogenesis in trout and mouse**. Expression profiles during spermatogenesis in trout and mouse (datasets GSE12769 and E-TABM-130) were compared. Mouse orthologs of differentially expressed trout genes were first identified using the Ensembl database and filtered to select only those genes with significant expression in at least one mouse developmental stage (Signal intensity ≥ median value). A correlation test was then performed to select genes with similar expression profiles (correlation ≥0.8) in trout and mouse developmental stages. Each line represents the expression signal of a trout clone and a corresponding mouse probeset. Gene expression in trout developmental stages, trout isolated germ cells, mouse developmental stages and trout isolated testicular cells is sorted according to the 9 trout expression clusters (A-I). Roman numerals (I-VIII) indicate trout developmental stages whereas numeric numbers (0-35) indicate mouse post-partum days. Samples used for the correlation test are indicated in red. Se = Sertoli cell; Sg = spermatogonia; Sc = spermatocytes; St = spermatids; Tu = mouse seminiferous tubules; Tt = mouse total testes. Log-2 transformed signal intensities are shown according to respective scale bars.

We next conducted a cross-species comparison to identify evolutionary-conserved genes that would also exhibit conserved expression during trout and mouse testis development. We used expression data corresponding to postnatal testis development in the mouse (GEO repository: GSE12769), as the first wave of spermatogenesis occurs, and compared the expression of 1105 differentially-expressed NR trout genes with their mouse orthologs. Firstly, we selected mouse genes with significant expression (≥ overall median value) in at least one developmental stage and identified 1044 genes that were differentially-expressed in trout and also consistently detected in mouse testes, suggesting roles during testis development or spermatogenesis for these genes in both species. In addition, we performed a correlation test to compare expression profiles in trout developmental stages (I, IIb, IIIb and V) and mouse post-partum ontogeny (0, 8, 18 and 30 dpp). This enabled us to select 442 clones (403 NR genes) that exhibit very similar developmental expression patterns and covered most of the expression clusters (Figure 5). Indeed, only genes in cluster C, whose expression profiles depend mainly on stage VIII gonads (a stage specific to highly cyclic fish species with no equivalent in mice), and in cluster I, which do not show important changes in expression during testis development, were found to exhibit low correlated expression with their mouse orthologs. Importantly, the genes selected by this filtration on the basis of developmental expression profiles also exhibited a clear-cut expression pattern in testicular cells isolated from mice. These genes might therefore be expressed in the same cell types in both species where they are likely to exert similar functions. Furthermore, the GO term analysis performed on 3 broad expression clusters (i.e. somatic, spermatogonia and meiotic/post-meiotic) for these conserved genes, not only confirmed previous results obtained for trout differentially-expressed genes, but extended these findings (Additional files 5 and 6). A striking example is that of the transforming growth factor receptor signalling pathway, for which 8 out of 9 differentially-expressed trout somatic genes were found to have conserved expression during mouse spermatogenesis (Bambi, Col1a2, Id1, Nfic, Nfix, Sptbn1a and b and Smad7). Similarly, 5 of the 6 genes involved in sperm mobility and/or fertilization that belong to the meiotic/post-meiotic cluster were also found conserved during mouse spermatogenesis (Gas8, HistH3, Klhl10, Pvrl3, Ropn1l and Sapg6). This confirms that identifying genes with evolutionary conserved expression is an efficient way to highlight factors with important functions in a given process.

Trout clones for which corresponding orthologous genes exhibit correlated expression or are significantly expressed during mouse spermatogenesis are labelled respectively "Correlated" and "Expressed" in additional file 1.

### Tissue profiling analysis reveals potential testis-specific genes in trout

**Figure 6:**
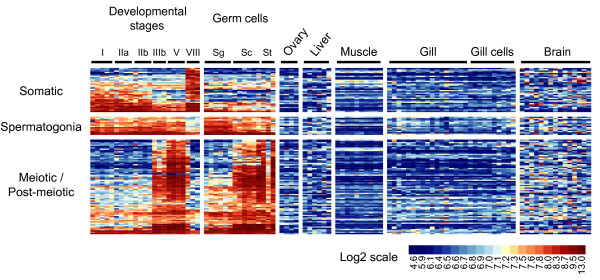
**Potential testis-specific genes as revealed by tissue-profiling analysis**. Genes were selected on the basis of their high expression (average signal intensity ≥3^rd ^quartile) in at least one testicular sample and low or null expression (average signal intensity < median) in 5 non-testicular tissues (Ovary, Liver, Muscle, Gill, and brain). Genes are displayed according to 3 broad expression clusters (i.e. somatic, spermatogonia and meiotic/post-meiotic). Roman numerals (I-VIII) indicate trout developmental stages. Sg = spermatogonia; Sc = spermatocytes; St = spermatids. Log-2 transformed signal intensities are shown according to the scale bar.

We used expression data from 5 non-testicular tissues (ovary, liver, muscle, gill and brain) hybridized on the same microarray to identify potential testis-specific genes and found 121 clones (111 genes) expressed predominantly in the testis with low or no expression in all the other tissues (Figure 6; labelled "Testis" in additional file 1). Only 23 of these potential testis-specific genes did not pass the standard deviation filtration step, thus demonstrating highly specific gene expression, not only at tissue level, but also at cellular level. Thirty four of these genes belong to somatic expression clusters and include notably 2 genes that are crucial for male gonad development: Dmrt1, a well-known sexually-dimorphic gene involved in both Sertoli cell differentiation and spermatogenesis maintenance [15], and Lhcgr, which encodes the receptor for LH and chorionic gonadotropins [16,17]. This also includes genes encoding receptors or extracellular proteins that may account for specific paracrine regulation within the testis (Ca6, Enpp6, Gpc3, Gpr175, Lgi1b, Lrp1, Podn, Tgfbr3 and Vmo1). The spermatogonia expression clusters also contain 14 genes with apparently preferential expression in the male gonad. Importantly, 6 of these genes - Cpsf1, Ddx19, Exosc8, Mcrs1, Msi2 and Piwil2, the latter gene encoding a stem-cell protein essential for germ cell differentiation [18] - were again found to bear functions related to RNA processing or transport. Finally 73 genes with expression detected in the testis only were found within the germline and meiotic/post-meiotic expression clusters: They consistently contained a number of genes coding for proteins known to be important in meiosis and/or spermatid development (Dmc1, Dnah9, Dnajb13, Dnal1, Gmcl1, Nek2, Nme5, Spata4, Trip13 and Zw10). In addition they contained genes encoding proteins localized on or involved in the mobility of flagella and cilia (c10orf63, c14orf143, Dnah8, Efhc1, Rshl3 and Ttll9) that are relevant sperm cell components. In addition, several transcription factors and cell cycle regulators that could be important for the progression throughout meiosis were found (Cdkn3, Lcmt1, Polr2i, Smc4, Styxl1 and Xccr3). It can be noted that when exploring enrichment for GO annotations linked to potential testis-specific genes, only terms related to meiosis or spermiogenesis were highlighted (Additional files 5 and 6), as previously observed in mammals [19].

Attention should be given to genes expressed in the female trout gonad as they are apparently also candidate genes for the male gonad function. Importantly, 67 additional genes highly expressed in the testis were also found to be expressed in the mature ovary (Additional file 7; labelled "Gonad" in additional file 1), including some genes already known to be important for meiosis or oocyte differentiation (Ccna1, Nmp2, Psmc3ip and Sycp3l). Also among this group of transcripts, a majority were annotated with functions related to cell proliferation or cell cycle regulation (Ada, Cdkn1c, Cks1b, Gsg2, Nasp and Pdcd6), spindle formation and chromosomal positioning or structure (Kntc2 and Smc2), DNA replication or repair (Cdt1, Mcm5, Nasp, Pold3, Recql5 and Rfc4) that are all of obvious interest for germ cell development.

### Candidate genes of interest in spermatogenesis regulation

**Figure 7:**
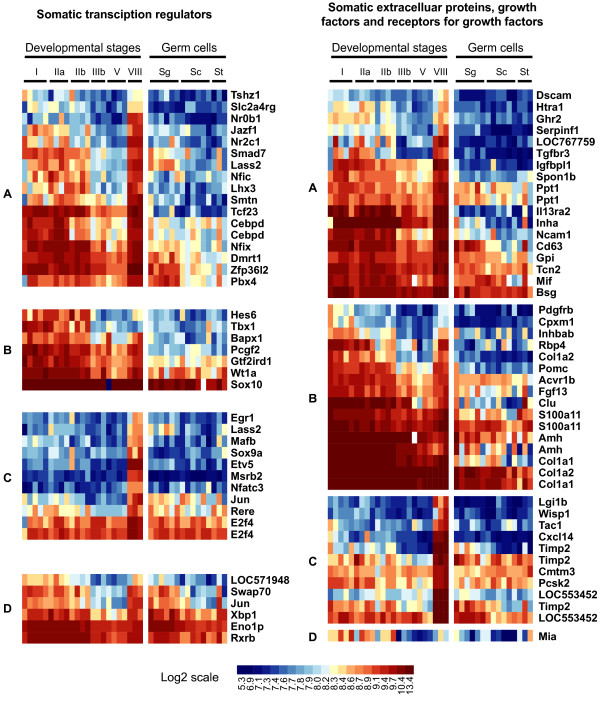
**Focus on candidate genes for spermatogenesis regulation**. Genes corresponding to GeneOntology terms "transcription factor activity" (GO:0003700) and "growth factor activity" (GO:0008083), "growth factor binding" (GO:0019838) or "extracellular space" (GO:0005615) were extracted from the somatic expression clusters previously identified. Genes are displayed according to 4 somatic expression clusters (A-D) and their corresponding gene names are given. Roman numerals (I-VIII) indicate trout developmental stages. Sg = spermatogonia; Sc = spermatocytes; St = spermatids. Log-2 transformed signal intensities are shown according to the scale bar.

To gain insight into the regulatory mechanisms that drive spermatogenesis we focused on genes preferentially expressed in the somatic cell compartment and may be involved in cell fate commitment during early testis maturation or in driving germ cell differentiation throughout the male reproductive cycle. This was performed by searching for genes encoding transcriptional regulators (corresponding to the following GO term "transcription factor activity"; GO:0003700), growth factors (growth factor activity; GO:0008083), receptors for growth factors (growth factor binding; GO:0019838 and "growth factor receptor activity" terms) or a more general class of extracellular proteins (extracellular space; GO: 0005615) in all 4 somatic cell expression clusters. Expression profiles of these genes are presented in figure 7.

**Figure 8:**
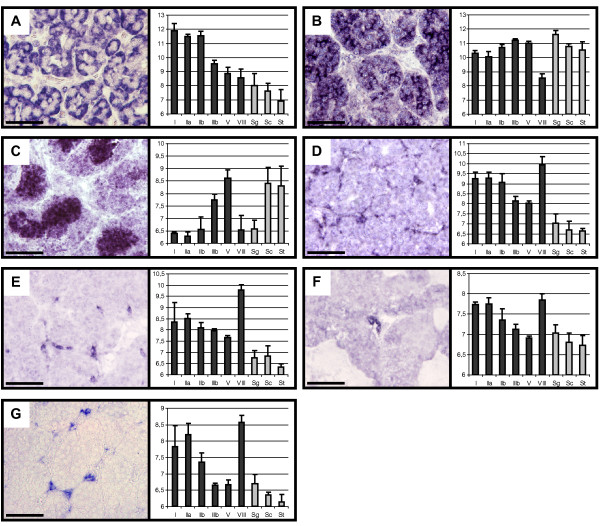
**Expression localisation of candidate genes**. *In Situ *Hybridization was performed on paraformaldehyde-fixed and paraffin-embedded trout testes using digoxigenin-labelled probes and NBT/BCIP for revelation. Scale bars indicate 50 μm. For each gene the corresponding expression signals (Log-2 transformed) as measured by microarrays are depicted. (A) Characteristic Sertoli cell staining for Amh (clone tcay0040.e.21) in immature trout testes. (B) Staining of spermatogonia for Ddx4 (clone 1RT40G14_C_D07) in stage II testes. (C) Detection of Txdnc6 (clone tcaa0002.a.02) in stage V testes spermatocytes and spermatids. (D) Expression of Tcf23 in peritubular cells (clone tcbk0026.d.10) as revealed in stage II testes. (E) Staining for Igfbp7 (clone tcbk0016.d.20) in Leydig cells from stage II testes. (F) Detection of Ghr2 (clone 1RT91I23_A_E12) in Leydig cells of stage VIII testes. (G) Tgfbr3 (clone tcbk0046.o.19) in Leydig cells of stage III testes.

We next performed in situ hybridization to localize expression for some of the somatic genes selected above. In order to validate our *In Situ *hybridization (ISH) protocol and the quality of the gonad samples we investigated the expression of genes of known cellular origin. The gene encoding the anti-Müllerian hormone, Amh, was strongly detected in Sertoli cells in the early stages I-II (Cluster C; Figure 8A) and, in agreement with its microarray expression profile, the staining progressively decreased in Sertoli cells in maturing testes and at the spawning stage (data not shown). The signal for Ddx4, the gene encoding the Drosophila VASA homolog, was strongest in spermatogonia and early spermatocytes (cluster F; Figure 8B) and decreased in subsequent germ cells in a similar way to the microarray experiment signals. Finally, the strong signal detected for Txdnc6 in both spermatocytes and spermatids (Figure 8C) confirmed its meiotic/post-meiotic profile (cluster H). Firstly, we investigated the expression of Tcf23 (Cluster A), a gene encoding the basic helix-loop-helix transcription regulator TCF23 (OUT), that we found to be preferentially-expressed in both the testis and ovary. Tcf23 staining was found at each developmental stage in thin cells very closely associated with spermatogenetic tubule borders (Figure 8D), which are likely to be peritubular cells. ISH performed on Igfb7, a gene encoding an Insulin-like growth factor-binding protein (Cluster A) revealed its expression in the interstitial Leydig cells (Figure 8E). In addition, Igfbp7 mRNA was also detected in endothelial cells from vessels (data not shown). Ghr2, a duplicated isoform of the gene encoding the growth-hormone receptor (Cluster A), was found to be expressed in stage VIII testes in large, round cells present in the interstitial tissue that are likely to be Leydig cells (Figure 8F). Finally, the expression of Tgfbr3, which encodes the type III TGF-beta receptor and that was only detected in the testis (Cluster A), was evidenced in interstitial Leydig cells in stage II testes (Figure 8G). Overall these results further confirmed our prediction of "somatic", "spermatogonial" and "meiotic/post-meiotic" expression clusters.

## Discussion

We have already applied preliminary large scale expression profiling (i.e., using 1K macroarrays) with the aim of studying the underlying molecular mechanisms of testis development at the beginning of the trout reproductive cycle [20]. In the present study we have confirmed and considerably extended previous findings, firstly by increasing the number of genes covered by the microarray, but also by refining the samples analysed, the gene annotation and the data-mining procedures. The use of testes at various developmental stages highlighted several gene activation or repression patterns throughout the reproductive cycle. In addition, using isolated germ cell populations we were further able to distinguish between somatic cell expression clusters and those corresponding to proliferating and differentiating germ cells. The identification of somatic genes was also reinforced by the use of spawning testes. Indeed, due to a dilution-effect resulting from the accumulation of germ cells, somatic gene expression decreased in maturing testes. On the contrary, in stage VIII gonads, where no more transcriptionnally active germ cells reside apart from a few spermatogonial stem cells, somatic genes are not diluted anymore. Moreover, this stage highlights somatic genes that undergo specific regulation as testes mature: low expression in stage VIII predicts their down regulation while high expression in stage VIII predicts their up regulation. We have thus reported the accurate spatio-temporal expression patterns for hundreds of testicular transcripts associated with the mitotic, meiotic and post-meiotic phases of germ cell development as well as with the somatic compartment of maturing testes in trout.

The establishment and description of transcriptomes underlying spermatogenesis have been mainly limited to a few mammalian species, especially in mice, rats and humans [21]. The broadening of such approaches to other vertebrates is important if we are to uncover the different regulatory pathways triggering tubular development, spermatogonial stem cell self-renewal, rapid proliferation and further differentiation of latter germ cells. In this respect, the cyclical and progressive features of trout spermatogenesis make it an original and interesting model for studying hormonal cues and paracrine communications sustaining germ cell development. In comparison to mammals where Sertoli cells sustain several generations of germ cells at the same time, the cystic organization and synchronous development of germ cell clones indeed make the trout testes a convenient way of studying cellular communications involved in the specific steps of spermatogenesis. The present dataset is likely to be of great utility in studying and identifying such networks. An important step forward will then be to understand how these networks are regulated and what factors (such as sexual steroids or other hormones) are responsible for the different expression profiles we have identified.

To gain a functional insight into the genes undergoing differential expression during the spermatogenetic process we undertook a sophisticated annotation procedure of trout clone cDNAs. This step is of particular importance when studying species such as trout for which the genome is not available and only little information exists in public databases (e.g. only 861 RefSeq entries in NCBI release 34 and 1920 entries in UniProtKB release 15.0 for *Oncorhynchus mykiss*). The starting point of our procedure was the use of expressed sequence tags (ESTs) instead of EST contigs that could lead to irrelevant annotations resulting from assembling errors. The high sequence coverage of trout clones, i.e. an average of 2.18 ESTs per clone, allowed us to map most of the corresponding transcripts on the genome of a closely related organism. For instance, up to 90% of the trout clones were linked to a potential fish orthologous gene in at least one of the 4 fish genomes. When this first mapping step was achieved, the use of mammalian orthologs and associated GO terms as provided by the Ensembl database allowed us to improve the somehow poor annotation of fish genomes. We believe that this annotation pipeline is more accurate than directly mapping trout sequences onto phylogenetically distant species and that such a strategy should be used in non-model species for which few genomic resources exist. As a matter of fact, the different expression patterns we conferred to the somatic compartment of the testis, the proliferation phase of germ cells or their subsequent differentiation, were consistently associated with annotations that are in agreement with what we know about these different testicular cell types and their biology. This further validated the biological significance and our interpretation of identified expression clusters. Obviously, some of the inferred orthologs and ontologies may still be erroneous, due to either partial, supernumerary duplications in fish genomes [22,23] or simply incorrect orthological relationships and annotations provided by the Ensembl database. Furthermore, even for accurately annotated and true orthologous genes, the biological processes they are involved in may differ from one organism to another. However, the use of statistical tests to investigate the content of expression clusters bypasses these issues by highlighting only relevant, either enriched or depleted, GO terms.

The search for mammalian trout gene orthologs was also carried out with the aim of comparing underlying expression programs in testis development and spermatogenesis in fish and mice. Genes that exhibit similar expression patterns in different species are very likely to play key roles in a given process. Their expression conservation indeed suggests that evolutionary forces act strongly on promoter regulatory regions and corresponding transcriptional regulators so that the encoded proteins achieve their functions both in a specific cell type and at a precise developmental stage. By comparing trout and mouse expression data we were able to identify genes not only expressed in the testes of both species but that display similar developmental expression patterns. These included genes more likely to be expressed in the germline and that tend to accumulate during the course of the spermatogenetic process, but also somatic genes that exhibit decreasing expression from puberty onwards as a consequence of their relative dilution. Here again the annotation was of great importance to compare expression profiles of accurate pairs of genes. From this point of view, it is important to note that the subgroup of correlated genes contained a number of genes with one-to-many or many-to-many orthologous relationships. Whereas this category of orthologs may lead to false correlated genes is still a possibility. We also believe that for such ambiguous sequence-based orthologs, expression conservation is a relevant way of identifying true orthologs at a functional level. The complement of conserved genes we have reported here is therefore likely to contain key factors fulfilling important functions during spermatogenesis that have been maintained throughout evolution and deserve special attention. On the other hand, the absence of correlation observed for a number of genes may underscore original pathways that may account for specific regulations in each species. It could also reflect species-specific features of spermatogenesis such as morphological organization and spatio-temporal development of the testis, and may thus represent molecular events that are difficult to detect or to observe in other species. However, it remains hard to distinguish between genes exhibiting true diverging expression profiles from discrepancies due to either wrong orthological relationships or artefacts due to the different technologies used to produce the two datasets.

Whereas several aspects of spermatogenesis such as events associated with meiosis and spermiogenesis are well conserved across species, testis organization and biology differ on certain points between amniote (reptiles, birds, mammals) and anamniote (fishes and amphibians) vertebrates [8]. Importantly, the number of spermatogonial generations, i.e. the number of mitoses spermatogonia undergo before meiosis, and the signalling regulating their differentiation is more specific to each species. In teleost fish testes, the molecular mechanisms triggering testis maturation are still poorly understood. A few growth factors that are produced by Sertoli cells and regulate germ cell proliferation and fate have however been identified. These include the TGF family members activin [24], anti-Müllerian hormone (AMH) [25] and gonadal soma-derived factor (GSDF) [26], as well as an ortholog of the platelet-derived endothelial cell growth factor (PD-ECGF) [27]. Importantly, Amh expression was found to be inhibited by 11-ketotestosterone, a teleost fish-specific androgen, and this steroid-mediated suppression of AMH action was sufficient to allow full completion of spermatogenesis in the eel [25]. This led AMH to be considered as a spermatogenesis-preventing substance and suggested that the initiation of meiosis in fish may occur through the suppression of an inhibitory activity rather than direct activation. In this regard, the cluster of somatic genes exhibiting apparent down regulation during the reproductive cycle, which includes the Amh and Inhba genes themselves, is of peculiar interest (cluster B). In an attempt to discover further factors that may sustain testicular maturation and could regulate spermatogonial cell proliferation and the onset of meiosis, we focused on those genes encoding extracelullar and transcription factors within the somatic expression clusters. In addition to the aforementioned genes Amh and Inhba, several well-known genes with important implications in testis development or spermatogenesis could be found: Dmrt1 and Sox9a are orthologs of mouse genes expressed in Sertoli cells and encode key transcription factors for testis differentiation [28,29]; Etv5 encodes a transcription factor, ERM, of which the expression in adult Sertoli cells controls spermatogonial stem cell self-renewal in mice [30]; Inha encodes a TGF family member that inhibits spermatogonia proliferation [31] and FSH production by the pituitary gland through a negative regulatory loop. Furthermore, we investigated the expression of previously uncharacterized factors in trout testes. Ghr2 encodes an isoform of the growth hormone (GH) receptor and was found to be expressed in Leydig cells. This localization is consistent with our finding that GH regulates steroidogenesis around spawning [12,32]. This factor could also take part in the local production of Insulin-like Growth Factor 1 (IGF1), which in turn might regulate the proliferation and/or differentiation of spermatogonia as previously proposed [33-35]. In addition, we have demonstrated that Igfbp7, which encodes an IGF-binding protein, is expressed in Leydig cells. This factor could therefore control the availability of other secreted factors and thus modulate germ cell proliferation. IGFBP7 is indeed a strong growth suppressor that can bind IGF1 and IGF2 as well as activin [36,37]. Furthermore, IGFBP7 was shown to inhibit activin-enhanced FSH-stimulated expression of Cyp19a1 and to suppress estradiol production in rat granula cells [38]. This suggests that IGFBP7 could similarly control the local production of steroids in the testis.

The large number of differentially-expressed genes we have reported here, i.e. almost 46% of the genes analysed, is most likely to reflect the important changes in cellular composition throughout spermatogenesis and the wide variety of underlying molecular and cellular functions in this process. In particular, germ cells undergo an extraordinary cellular differentiation process and fulfil unique functions in the organism. Their expression program therefore significantly differs from that of somatic cells and notably involves the expression of many testis specific gene products. To address this issue in trout, we used expression data from 5 non-testicular tissues to identify transcripts only detected in the testes or the gonads. Not surprisingly, most of these genes were expressed within the germline, especially in the meiotic and post-meiotic germ cells. However, a few of them were also found in somatic expression clusters, notably including Dmrt1 and Lhgcr, and may be involved in original pathways leading to successful sustaining of germ cell development. We investigated the expression of such a gene, Tgfbr3, and found it was expressed in Leydig cells. Tgfbr3 encodes the Transforming growth factor (TGF)-beta receptor type III (TGFBR3 or betaglycan), which orchestrates the TGF-beta superfamily signalling by binding both BMP and TGF-beta members [39]. Notably, TGFBR3 was shown to function as an inhibin co-receptor with activin receptor type II (ActRII) and to mediate functional antagonism of activin signalling by conferring inhibin sensitivity [40]. Betaglycan might thus play an important role within the testis in regulating paracrine actions of activin and inhibin produced in response to gonadotropes. We also demonstrated that Tcf23, a gene detected only in gonads from both sexes, was expressed by peritubular cells. Tcf23 encodes the basic helix-loop-helix factor TCF23 (also called OUT), a transcriptional repressor involved in myoblast differentiation in mammals [41], for which expression in testicular contractile cells is thus relevant. Tissue-specific expression is often correlated with important physiological roles for a gene in that tissue and the testis and gonad specific genes we identified may have important implications during spermatogenesis or testis development. Besides, these testis-specific genes are also of great interest to identify common cis DNA regulatory elements responsible for their restricted expression in the male gonad.

## Conclusion

In this study we investigated the underlying expression program in trout testis development and spermatogenesis using cDNA microarrays. In addition to genes preferentially expressed in germ cells at each differentiation stage, our expression profiling analysis has revealed several distinct somatic gene expression clusters that may sustain germ cell survival and testis maturation throughout the male reproductive cycle. Furthermore, to point out and provide the community with the most promising and important genes, we identified potential trout testis-specific genes as well as evolutionary-conserved genes involved in spermatogenesis in vertebrates. In addition to the accurate annotation of trout genes represented on the microarray, our dataset represents a valuable and solid base for further investigating the molecular mechanisms involved in testis differentiation and the onset of spermatogenesis in fish.

## Methods

### Animals and sampling

Male rainbow trout (*Oncorhynchus mykiss*) were obtained from the INRA experimental fish farm (Drennec, France) where they were maintained under natural conditions of light and photoperiod at 10°C under constant photoperiod. Experimental research on animal reported here was performed in conformity with the principles for the use and care of laboratory animals in compliance with French and European regulations on animal welfare. Furthermore experimenters were delivered an authorization given by the French "Direction des Services Vétérinaires" to conduct or supervise experimentations on live animals. Sampling was performed at several times throughout the complete annual reproductive cycle (see below). Testes were recovered, weighed to determine the gonadosomatic index (GSI) and tissue were rapidly immersed in paraformaldehyde or Bouin's solution for further histological analyses, or frozen until RNA extraction. Accurate determination of testicular developmental stages was achieved by combining histological analysis of Bouin's solution fixed samples (on the basis of the most differentiated germ cell type present in the gonad) and GSI, as previously described in details [12,42].

The samples retained for the microarray experiment included: - testes in early immature stages (February-March) containing either exclusively slowly-dividing type A spermatogonia (Stage I, n = 5); - rare (Stages IIa, n = 4) or growing numbers (Stage IIb, n = 4) of actively-dividing type B spermatogonia - testes in early maturation stages (April-May) also containing large numbers of meiotic spermatocytes (Stage IIIb, n = 3) and post-meiotic spermatids (Stage V, n = 4) - finally, fully mature spawning testes producing milt (October) and containing essentially mature spermatozoa (stage VIII, n = 3).

### Germ cell isolation

Germ cell isolation procedures were adapted from previously described protocols [43]. Briefly, pooled testes from immature males (stages I-II) or from individuals in early maturation staging (stages III-IV) animals were minced into small pieces and digested using collagenase (2.2 mg/ml) in Ca2+ free Hank's medium for 6 hours at 12°C, under gentle agitation. Cell suspensions were rinsed in L15 medium and gently shaken overnight in L15 medium with 1% BSA. Cell suspensions were allowed to sediment, the supernatants were discarded and the cell pellets were gently homogenized in L15 medium with 1% BSA using a Dounce homogenizer. The resulting cell suspensions were filtered through nylon gauze (150 μm pore size) and subjected to an additional homogenization in a Dounce homogenizer. The cell suspensions were filtered again using nylon gauze (50 and 20 μm pore sizes) and pelleted by centrifugation at 50 g for 10 and 5 minutes. The cell pellets were resuspended in L15 medium with 1% BSA, loaded onto 90% percoll gradients and centrifuged for 40 min at 500 g and 5 min at 50 g. The upper floating layers were recovered, rinsed in L15 medium with 1% BSA and filtered using nylon gauzes (20 μm pore size) prior to centrifugal elutriation (JE5 Beckman Instruments). Cell separation was performed at constant rotation speed (2000 rpm) and increasing flow rates (6.6, 7.8, 8.6, 9.5, 10.5, 13.5, 13-15.1, 19 and 21 ml/min) in L15 media with 1% BSA. Collected fractions were rinsed and pelleted. An aliquot was transferred in Trizol reagent and stored at -80°C for RNA extraction and transcriptome analysis (see below). An aliquot of each fraction was also used for microscopic observation after fixation in Bouin's solution, double inclusion in agar then parrafin, 4 μm sectioning and trichrome coloration. Spermatogonia-enriched samples (n = 6) were obtained by pooling 19 and 21 ml/min elutriation fractions obtained from immature fish. They contained 30 to 90% type-A spermatogonia and 70 to 0% type-A and type-B spermatogonia, and included less than 10% Sertoli cells. Spermatocyte-enriched samples (n = 6) correspond to 8.6 - 9.5 ml/min fractions obtained from mature gonads. They contained ≥70% primary spermatocytes, contaminated with B spermatogonia or secondary spermatocytes. Spermatid-enriched samples (n = 3) correspond to 6.6 and 7.8 ml/min pooled elutriation fractions and contained ≥90% post meiotic cells.

### cDNA microarray experiment

#### cDNA microarray construction

cDNA microarrays were generated using CRB GADIE http://www-crb.jouy.inra.fr/ as previously described [44]. Briefly, 9023 rainbow trout cDNA clones from pooled-tissue libraries [45,46] were amplified by PCR using vector-specific primers. PCR products were evaporated, resuspended in water and spotted onto nylon membranes (7.6 × 2.6 cm; Nybond-N+, Amersham Biosciences) using a Biorobotics MicroGrid-II arrayer (Genomics Solution). Spotted membranes were then denatured (150 mM NaOH, 1.5 M NaCl), neutralised (1 M Tris-HCl, pH 7.5, 1.5 M NaCl), washed (2× SSC) and fixed (80°C for 2 hours, UV 120000 μJ).

#### RNA extraction and cDNA target synthesis

Total RNA was extracted from either entire gonads or isolated germ cells using Trizol reagent (Invitrogen), quantified on a NanoDrop ND-1000 (Thermo Scientific) and quality-controlled on a Bioanalyser 2100 (Agilent). For cDNA target labelling, 5 μg of RNA were reverse-transcribed for 2 hours at 42°C in presence of radiolabelled dNTP (30 μCi [alpha-33P] dCTP, 120 μM dCTP and 20 mM dATP, dTTP and dGTP) using an oligo(dT) primer and 400 units of Superscript II reverse transcriptase (Invitrogen) in a total volume of 46 μl. RNA was degraded at 68°C for 30 min with 1 μl of 10% SDS, 1 μl of 0.5 M EDTA and 3 μl of 3 M NaOH. The reaction was then equilibrated (room temperature, 15 minutes) and neutralised (10 μl of 1 M Tris-HCI and 3 μl of 2 N HCl).

#### Microarray hybridization and raw data production

Prehybridization of microarrays was performed in a hybridization buffer (5× Denhardt's, 5× SSC, 0.5% SDS) at 65°C for 4 hours. Labelled cDNA targets were denatured at 95°C for 5 minutes and incubated with microarrays for 48 hours at 65°C in the same buffer. Microarrays were then washed three times for 1 hour at 68°C (0.1× SSC, 0.2% SDS) prior to exposure to phosphor-imaging plates for 65 hours. Plates were finally scanned using a FUJI BAS 500 and signal acquisition was performed using BZscan software [47]. Each microarray was also hybridized (but at 42°C) with a 33P-labelled oligonucleotide (TAATACGACTCACTATAGGG) that recognizes a vector sequence in every PCR product to quantify the amount of cDNA present in each spot.

#### Normalisation procedure

Raw expression data were first corrected for the amount of spotted cDNA by dividing the sample signal of each spot by its corresponding vector signal (Si/Vi) [48]. This procedure is efficient in correcting between-array spotting differences but also simultaneously biased results relative to gene expression levels: sample signals corresponding to low vector signals are indeed artificially increased in comparison to those with high vector signals which are minimized. To avoid this bias, the corrected sample signal of each spot was further multiplied by the median vector signal of this same spot on all the arrays ((Si/Vi) × medVi). Expression values were then Log-2-transformed and subjected to a quantile-quantile normalisation [49] using AMEN software http://sourceforge.net/projects/amen/[50]. Raw data as well as a normalised expression files are available on the Gene Expression Omnibus (GEO) repository (http://www.ncbi.nlm.nih.gov/geo/; GSE16029).

#### Statistical and cluster analyses

Overall similarities in gene expression across all samples were first computed by means of supervised hierarchical clustering using Cluster (uncentered correlation, average linkage) and the corresponding dendogram was generated using TreeView [51].

Non-informative clones for which the quantity of cDNA spotted was too low (oligonucleotide signal < 3 times the background level in more than 20% of samples) were removed prior to statistical analyses. A permutation test (F's statistic, FDR ≤5‰) was used to identify differentially-expressed transcripts during spermatogenesis by comparing all sample groups to each other (Stage I, n = 5; stage IIa, n = 4; stage IIb, n = 4; stage IIIb, n = 3; stage V, n = 4; stage VIII, n = 3; spermatogonia, n = 6; spermatocyte, n = 6; and spermatid, n = 3) as previously described [19,52] using AMEN software. Highly differentially-expressed genes were selected on the basis of their standard deviation (SD ≥ 0.4) from the median expression of selected samples (Stages I, IIIb, V, VIII, spermatogonia, spermatocytes and spermatids). Differentially-expressed transcripts were subjected to the PAM clustering algorithm using AMEN software and grouped into 9 expression clusters according to both developmental stages and isolated germ cell fractions. PAM algorithm was performed using "standardized" signal intensities, so that genes were classified according to their relative expression profile. However for heatmap representations, "non-standardized" signals were used to range genes according to their expression level within each cluster using the hierarchical classification algorithm. This heatmap representation is more informative as it accounts for the range of dynamic expression of genes as well as for their co-regulation.

#### Tissue-profiling analysis

Expression data corresponding to normal adult ovary, liver, muscle, gill and brain samples hybridized on the same microarray platform were corrected as described above. All expression files were then merged and subjected to a quantile-quantile normalization step. The median cell type and tissue replicate expression values were computed and an empirical approach was used to identify preferentially-expressed transcripts in each tissue. A transcript was considered expressed predominantly in a given tissue if its normalized expression signal was in the 25% highest (≥3^rd ^quartile) and in the 50% lowest (< median) in all other tissues. Of note is that the median signal intensity of a microarray is a commonly accepted estimation of the background and that expression signals under this value can thus be considered as not expressed or at least not detected.

### Functional data mining

#### Trout cDNA annotation strategy

All 20008 ESTs corresponding to the 9023 clones used for the microarray were obtained http://www.sigenae.org and mapped on the masked genome (RepeatMasker program) of 4 model fish species (Danio rerio, Gasterosteus aculeatus, Orizias latipes and Takifugu rubripes) using the BLAT algorithm [53] in RNA, DNA, translated RNA and translated DNA modes (minimal score ≥30 and identity ≥60%). For each EST, the best matches (best score plus scores ≥0.95* [best score]) on each genome were retained (Additional file 8). Each match was further associated with exonic, intronic or intergenic regions of existing genes (Ensembl gene ID) using the "ensGene.txt" file provided by the UCSC Genome Browser http://genome.ucsc.edu/[54,55]. The corresponding Ensembl protein family ID and the Ensembl gene IDs of Fish orthologs were also extracted using the Ensembl BioMart (version 52; http://www.ensembl.org/index.html; [56,57]). The genes matched by each EST in the 4 fish species were then compared and the number of times that orthologous genes or genes from the same protein family were found was counted (Confidence index "n" in additional file 1). Each clone was then annotated with the EST exhibiting the highest confidence index "n". For these genes, the corresponding gene name, description, and their mouse, rat and human orthologs were also extracted from the Ensembl database. Only clones for which the retained EST exhibited a confidence index "n" ≥2 or that matched at a distance of ≤500 base pairs from a predicted Ensembl gene with a BLAT score ≥50 were further considered (Annotation status labelled "confident" in additional file 1).

#### GeneOntology term analysis

GO terms ("biological process", "molecular function" and "cellular component") associated with all retained Ensembl genes as well as their fish, rat, mouse and human orthologs were extracted using the Ensembl BioMart (Ensembl version 52) and associated with the corresponding trout clones. Functional mining of expression clusters was then performed by searching for overrepresented GO terms (Gaussian hypergeometric test) in comparison with the whole set of well-measured genes using AMEN software. To avoid gene redundancy and as the search for trout orthologs was performed in 4 different species, only one Ensembl gene ID was returned for each clone, using the following order of preference depending on the information available for the species: Gasteosteus aculeatus, Danio rerio, Oryzias latipes or Takifugu rubripes. Ambiguously annotated clones for which the corresponding EST mapped several genes were not considered. GO terms were considered as overrepresented when the p-value was ≤10^-6 ^and ≥3 non-redundant genes bearing this term were found within the corresponding cluster. To avoid redundancy between closely related terms an Ontology Specific Information Rate (OSIR [50] cut-off of ≥0.95 was selected).

### Cross-species comparison

For each fish Ensembl gene ID, the mouse ortholog Ensembl gene ID(s) and corresponding Affymetrix GeneChip probeset(s) (Mouse Genome 430 2.0 Array) were extracted from the Ensembl database (version 52; Additional file 1). Mouse data for postpartum testis development (GEO repository: GSE12769) and isolated testicular cells [19](ArrayExpress repository: E-TABM-130) were downloaded and pre-processed using the RMA normalization module with AMEN software. Sample replicates were averaged and the following filtration procedure was applied: Mouse genes with significant testicular expression levels (Signal ≥ background expression cut-off (overall dataset median)) in at least one developmental stage and orthologous to trout differentially-expressed genes were first selected. Among them, genes exhibiting highly similar expression patterns (correlation coefficient ≥0.8) between trout (developmental stage I, IIb, IIIb and V) and mouse (day postpartum 0, 8, 18 and 30) were then selected.

### *In situ *hybridisation

Bacterial clones were obtained from the CRB GADIE resource centre (Jouy-en-Josas, France) or the USDA (Washington D.C., USA): tcay0040.e.21 (Amh), 1RT40G14_C_D07 (Ddx4), tcaa0002.a.02 (Txndc6), tcbk0026.d.10 (Tcf23), tcbk0016.d.20 (Igfbp7), 1RT91I23_A_E12 (Ghr2) and tcbk0046.o.19 (Tgfbr3). Briefly, clones were grown in LB-ampicillin medium, plasmids were extracted and cDNA inserts were amplified by PCR using vector-specific primers. PCR products were purified and used as templates for digoxigenin (DIG)-labeled probe synthesis using the Riboprobe^® ^Combination system - T3/T7 RNA polymerase (Promega).

*In Situ *Hybridization experiments were performed on 5 μm sections of paraformaldehyde-fixed and paraffin-embedded trout testes. After dewaxing and rehydration, all the following steps were performed using the InsituPro VS automate (Intavis Bioanalytic Instruments). Briefly, sections were post-fixed (paraformaldehyde 4%, 20 min), permeated for 20 min at 37°C with 1 μg/mL Proteinase K, for 10 min in 50 mM glycine and post-fixed again (paraformaldehyde 4%, 10 min). Slides were then pre-hybridized for 2 hours at 60°C (formamid 50%, 2× SSC, 1× Denhardt, dextran sulfate 10% and yeast tRNA at 250 μg/mL) and hybridized for 12 hours at 60°C with 2 ng/μL of DIG-labeled cRNA probes in the same buffer. After hybridization, the sections were treated for 30 min at 37°C with RNase (10 μg/mL in 2× SSC), washed successively at 37°C in 2× and 0.1× SSC, coated for 40 min in 2% sheep serum and incubated for 2 hours with alkaline phosphatase-conjugated anti-DIG antibody (Roche Applied Science; 1:1000) in 2% sheep serum. Slides were finally rinsed, revealed for 12 hours with NBT/BCIP and mounted in Mowiol solution.

## Authors' contributions

ADR, JJL, RH, FC and FLG designed research; ADR, ASG, JM, MJR, DE, KH performed research; ADR, JM, RH, FC and FLG analyzed data; and ADR and FLG wrote the manuscript. All authors have read and approved the final version of the manuscript.

## Supplementary Material

Additional file 1 **Differentially-expressed genes during trout spermatogenesis**. A searchable excel file containing normalized expression data (Log-2 transformed), annotations, and information about "Expression cluster" (A to I), "Tissue-profiling" ("Testis" and "Gonad") and "Expression conservation" with mouse ("Not detected", "Expressed" or "Correlated") for all differentially-expressed trout clones. Annotation information provided contains the "Clone Name", the annotation status ("None", "Not confident", "Confident"), the "EST Name" retained for annotation, the corresponding "BLAT Score", "Matching type" ("Exonic", "Intronic" or "Intergenic"), the "Overlapping length" (base pairs; if < 0 corresponds to the distance from the closest gene) and the confidence index "n", the "original matched Ensembl gene ID", the corresponding "Gene Symbol" and "Description", the "Fish ortholog Ensembl gene IDs" and "Fish ortholog descriptions", the "Non redundant ID" (Ensembl Gene ID of fish orthologs according to the following species availability: Gasteosteus aculeatus, Danio rerio, Oryzias latipes or Takifugu rubripes), the "Mammalian ortholog Ensembl gene IDs" (for human, mouse and rat), the "Mammalian ortholog gene symbols" and "Mammalian ortholog gene descriptions" and the corresponding mouse "Affymetrix ProbeSets". Click here for file

Additional file 2 **qPCR validation of microarray expression profiles**. Total RNAs (2 μg, DNAse-treated) were submitted to reverse-transcription (RT) using random hexamer primers and MMLV reverse transcriptase for 2 hours at 37°C. Real-time PCR assays were performed on the StepOne™ Real-Time PCR System (Applied Biosystems) using 1:120 diluted RT products and the Fast SYBR^® ^Green Master Mix (Applied Biosystems). The amplification program consisted of an initial denaturation at 95°C for 20 seconds; 40 cycles of 95°C for 3 seconds, 60°C for 30 seconds; and a final progressive increase of temperature (From 65°C to 90°C, 0.5°C/second) for melting curve analysis. Cycle threshold (Ct) was manually setup and relative expression levels were normalised using an empirically designed reference gene, Rps15 (clone 1RT58B15_B_A08). Efficiency (95-105%) of PCR amplification was verified using serial dilutions of pooled RT products and the melting curve analysis was performed at the end of each real time PCR assay to control for specificity. Stage effects were determined using a non-parametric ANOVA (Kruskall-Wallis test). Forward (FW) and reverse (RV) primers were as follows: Amh (FW-GGGAATAACCATGCTATCCTGCTTAA; RV-CTCCACCACCTTGAGGTCCTCATAGT), Dmrt1 (FW-GGACACCTCCTACTACAACTTCTA; RV-GTTCGGCATCTGGTATTGTTGGT), Rps15 (FW-CCTGGGGGAGTTCTCTATCACCT; RV-GGGATGAAACGGGAAGAATGTGT), Slc26a4 (FW-CGGCACAAACATATACAGGAA; CCACCGTGACTCTCAATCGTTCT), Sox9a (FW-GTATTTCCAGTTCTTTCAGCCA; RV-TTTGCTATCTAGTTGTGTACGG), Sox9b (FW-AGCAGCAGTTGGATTCTAAAGTC; RV-ACACTTCTCCTGTTCGTCTG), Tbx1 (FW-CTTCGGCTACTAGTGCTGTGGAA; RV-CAACCTCCCAACCTTCTAACCTC). Roman numerals (I-VIII) indicate testicular developmental stages. GA and GB = type A and type B spermatogonia, repsectively; Sc = spermatocytes; St = spermatids. Log-2 transformed signal intensities from microarrays are also shown (Mean+-SD, left panels). Click here for file

Additional file 3 **Functional mining ("molecular function" and "cellular component") of trout spermatogenetic clusters**. Over-represented "molecular function" and "cellular component" terms from the GeneOntology (GO) were identified in the 9 expression clusters shown in Figures 2 and 3 (A-I). Rectangles indicate the observed (left) and expected (right) numbers of genes bearing the corresponding GO term whereas the number of genes exhibiting this GO term on the entire microarray is given on the left. Only GO terms with a p-value ≤ 10^-6 ^and for which at least 3 non-redundant genes belonged to the cluster were considered as statistically-enriched. To avoid redundancy between closely related terms an Ontology Specific Information Rate (OSIR) cut-off ≥0.95 was selected. Numbers in bold indicate a statistical enrichment for a given GO term according to the scale bar. Click here for file

Additional file 4 **Enriched GO terms associated with trout spermatogenesis expression clusters**. An Excel file containing all enriched GeneOntology terms ("Biological process", "Molecular function" and "Cellular process") for the 9 clusters shown in Figures 2 and 3. Only GO terms with a p-value ≤ 10^-6 ^and for which at least 3 non-redundant genes belonged to the cluster were considered as statistically-enriched, as previously mentioned. A low Ontology Specific Information Rate (OSIR) cut-off ≥0.05 was selected to allow redundancy between closely related terms. Click here for file

Additional file 5 **Functional mining of trout spermatogenetic, evolutionary conserved and testis-specific expression clusters**. Enriched "biological process", "molecular function" and "cellular component" GeneOntology terms in "somatic" (Clusters A-D), "spermatogonial" (Clusters E and F) and "meiotic/pot-meiotic" (Clusters H-I) expression clusters as evidenced in 3 conditions: - genes differentially expressed during spematogenesis in trout; - subgroup of genes with correlated expression during mouse spermatogenesis; and - subgroup of genes exhibiting testis-specific expression. Rectangles indicate the observed (left) and expected (right) numbers of genes bearing the corresponding GO term whereas the number of genes exhibiting this GO term on the entire microarray is given on the left. Only GO terms with a p-value ≤ 10^-6 ^and for which at least 3 non-redundant genes belonged to the cluster were considered as statistically-enriched. To avoid redundancy between closely related terms an Ontology Specific Information Rate (OSIR) cut-off ≥0.95 was selected. Numbers in bold indicate a statistical enrichment for a given GO term according to the scale bar. Click here for file

Additional file 6 **Enriched GO terms in trout spermatogenetic, evolutionary conserved and testis-specific expression clusters**. An Excel file containing all enriched GeneOntology terms ("biological process", "molecular function" and "cellular component") in "somatic" (Clusters A-D), "spermatogonial" (Clusters E and F) and "meiotic/pot-meiotic" (Clusters H-I) expression clusters as evidenced in 3 conditions: - genes differentially expressed during spematogenesis in trout; - subgroup of genes with correlated expression during mouse spermatogenesis; and - subgroup of genes exhibiting testis-specific expression. Only GO terms with a p-value ≤ 10^-6 ^and for which at least 3 non-redundant genes belonged to the cluster were considered as statistically-enriched, as previously mentioned. A low Ontology Specific Information Rate (OSIR) cut-off ≥0.05 was selected to allow redundancy between closely related terms. Click here for file

Additional file 7 **Tissue-specific gene expression in trout**. Tissue-specific genes were identified on the basis of their high expression (average signal intensity ≥3^rd ^quartile) in at least one tissue (Testis, ovary, Liver, Muscle, Gill, or brain) and low or no expression (average signal intensity < median) in the 5 other tissues. Testis and Gonad specific genes are displayed according to 3 broad spermatogenesis expression clusters (i.e. somatic, spermatogonia and meiotic/post-meiotic). Log-2 transformed signal intensities are shown according to the scale bar. Click here for file

Additional file 8 **Trout clone annotation strategy**. An ideogram presenting the annotation strategy for annotating a trout cDNA clone. BLAT alignments were performed on the fish species genomes available at the UCSC Genome Browser http://genome.ucsc.edu/. Annotation was performed using the Ensembl database http://www.ensembl.org/index.html version 52. Click here for file
