# Supplementary material for: Expression profiling of rainbow trout testis development identifies evolutionary conserved genes involved in spermatogenesis
Source: BMC Genomics. 2009 Nov 20;10:546. doi: 10.1186/1471-2164-10-546 (PMC2786911; doi:10.1186/1471-2164-10-546)
Supplement: Additional file 2 — qPCR validation of microarray expression profiles. Total RNAs (2 μg, DNAse-treated) were submitted to reverse-transcription (RT) using random hexamer primers and MMLV reverse transcriptase for 2 hours at 37°C. Real-time PCR assays were performed on the StepOne™ Real-Time PCR System (Applied Biosystems) using 1:120 diluted RT products and the Fast SYBR® Green Master Mix (Applied Biosystems). The amplification program consisted of an initial denaturation at 95°C for 20 seconds; 40 cycles of 95°C for 3 seconds, 60°C for 30 seconds; and a final progressive increase of temperature (From 65°C to 90°C, 0.5°C/second) for melting curve analysis. Cycle threshold (Ct) was manually setup and relative expression levels were normalised using an empirically designed reference gene, Rps15 (clone 1RT58B15_B_A08). Efficiency (95-105%) of PCR amplification was verified using serial dilutions of pooled RT products and the melting curve analysis was performed at the end of each real time PCR assay to control for specificity. Stage effects were determined using a non-parametric ANOVA (Kruskall-Wallis test). Forward (FW) and reverse (RV) primers were as follows: Amh (FW-GGGAATAACCATGCTATCCTGCTTAA; RV-CTCCACCACCTTGAGGTCCTCATAGT), Dmrt1 (FW-GGACACCTCCTACTACAACTTCTA; RV-GTTCGGCATCTGGTATTGTTGGT), Rps15 (FW-CCTGGGGGAGTTCTCTATCACCT; RV-GGGATGAAACGGGAAGAATGTGT), Slc26a4 (FW-CGGCACAAACATATACAGGAA; CCACCGTGACTCTCAATCGTTCT), Sox9a (FW-GTATTTCCAGTTCTTTCAGCCA; RV-TTTGCTATCTAGTTGTGTACGG), Sox9b (FW-AGCAGCAGTTGGATTCTAAAGTC; RV-ACACTTCTCCTGTTCGTCTG), Tbx1 (FW-CTTCGGCTACTAGTGCTGTGGAA; RV-CAACCTCCCAACCTTCTAACCTC). Roman numerals (I-VIII) indicate testicular developmental stages. GA and GB = type A and type B spermatogonia, repsectively; Sc = spermatocytes; St = spermatids. Log-2 transformed signal intensities from microarrays are also shown (Mean+-SD, left panels). [file 1471-2164-10-546-S2.PDF]

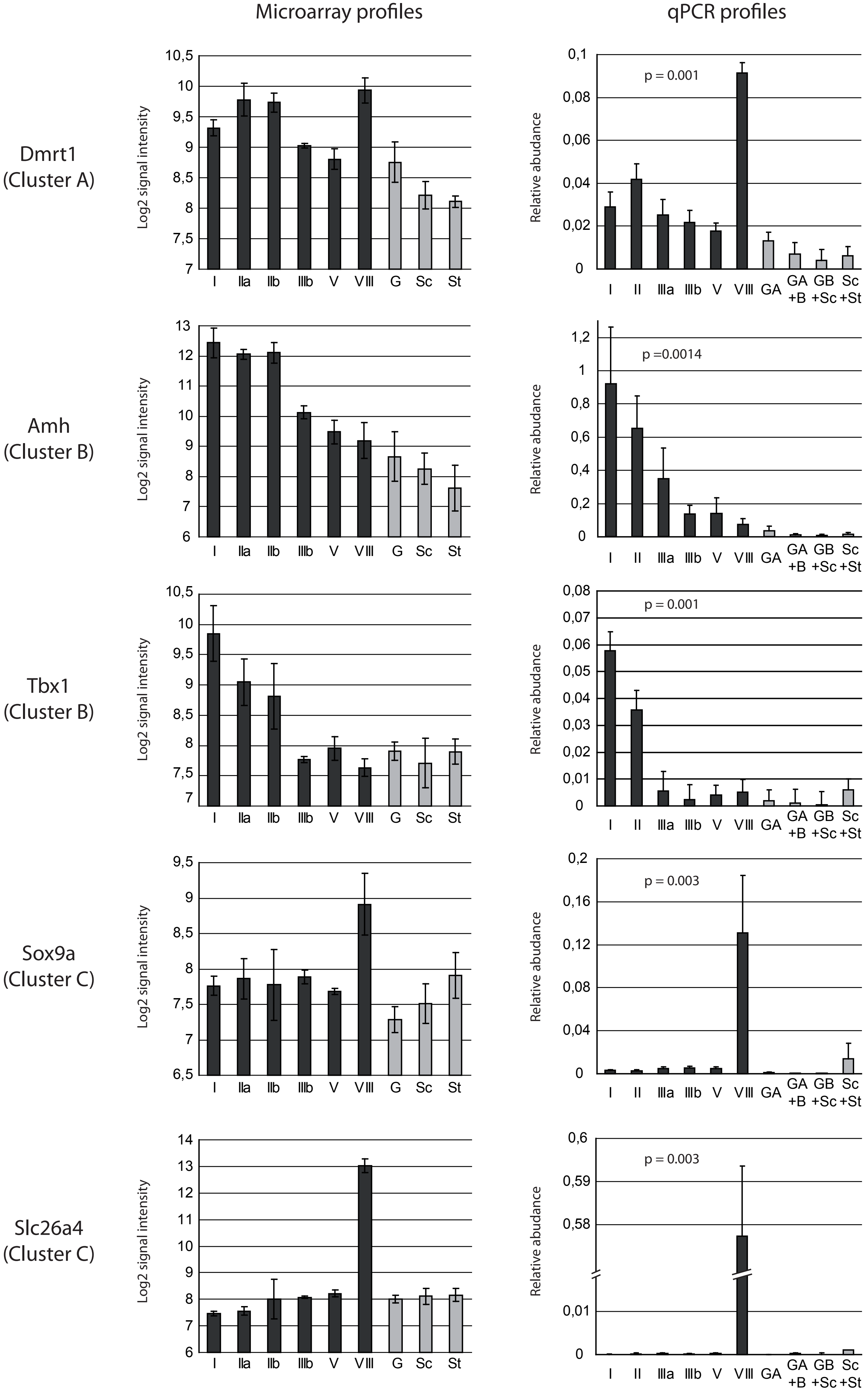
Microarray profiles
qPCR profiles
Dmrt1
(Cluster A)
Amh
(Cluster B)
Tbx1
(Cluster B)
Sox9a
(Cluster C)
Slc26a4
(Cluster C)
Log2 signal intensity
Relative abudance
p = 0.001
p =0.0014
p = 0.001
p = 0.003
p = 0.003
I
IIa
IIb
IIIb
V
VIII
G
Sc
St
I
II
IIIa
IIIb
V
VIII
GA
GA +B
GB +Sc
Sc +St
10,5
10
9,5
9
8,5
8
7,5
7
0,1
0,08
0,06
0,04
0,02
0
13
12
11
6
1,2
1
0,8
0,6
0,4
0,2
0,07
0,05
0,03
0,01
6,5
0,16
0,12
14
0,6
0,59
0,58
