# Supplementary material for: Expression profiling of rainbow trout testis development identifies evolutionary conserved genes involved in spermatogenesis
Source: BMC Genomics. 2009 Nov 20;10:546. doi: 10.1186/1471-2164-10-546 (PMC2786911; doi:10.1186/1471-2164-10-546)
Supplement: Additional file 5 — Functional mining of trout spermatogenetic, evolutionary conserved and testis-specific expression clusters. Enriched "biological process", "molecular function" and "cellular component" GeneOntology terms in "somatic" (Clusters A-D), "spermatogonial" (Clusters E and F) and "meiotic/pot-meiotic" (Clusters H-I) expression clusters as evidenced in 3 conditions: - genes differentially expressed during spematogenesis in trout; - subgroup of genes with correlated expression during mouse spermatogenesis; and - subgroup of genes exhibiting testis-specific expression. Rectangles indicate the observed (left) and expected (right) numbers of genes bearing the corresponding GO term whereas the number of genes exhibiting this GO term on the entire microarray is given on the left. Only GO terms with a p-value ≤ 10-6 and for which at least 3 non-redundant genes belonged to the cluster were considered as statistically-enriched. To avoid redundancy between closely related terms an Ontology Specific Information Rate (OSIR) cut-off ≥0.95 was selected. Numbers in bold indicate a statistical enrichment for a given GO term according to the scale bar. [file 1471-2164-10-546-S5.PDF]

Depleted — p-value — Enriched
1 0.95 0.05 1e-5 0

		Differentially expressed in the trout			Correlated with mouse			Testis Specific		
		Somatic	Gonia	Meiotic/ p-meiotic	Somatic	Gonia	Meiotic/ p-meiotic	Somatic	Gonia	Meiotic/ p-meiotic
**Biological Process**	4944	557	215	424	182	49	126	31	11	49
antigen processing and presentation	24	11 / 3	1 / 1	0 / 2	3 / 1	0 / 0	0 / 1	0 / 0	0 / 0	0 / 0
C21-steroid hormone metabolic process	10	6 / 1	1 / 0	1 / 1	1 / 0	0 / 0	0 / 0	0 / 0	0 / 0	0 / 0
positive regulation of cyclic nucleotide biosynthetic process	5	4 / 1	0 / 0	0 / 0	2 / 0	0 / 0	0 / 0	0 / 0	0 / 0	0 / 0
water homeostasis	3	3 / 0	0 / 0	0 / 0	0 / 0	0 / 0	0 / 0	0 / 0	0 / 0	0 / 0
glycosphingolipid catabolic process	3	3 / 0	0 / 0	0 / 0	1 / 0	0 / 0	0 / 0	0 / 0	0 / 0	0 / 0
cell-substrate junction assembly	3	3 / 0	0 / 0	0 / 0	2 / 0	0 / 0	0 / 0	0 / 0	0 / 0	0 / 0
oxygen transport	10	8 / 1	0 / 0	0 / 1	7 / 0	0 / 0	0 / 0	0 / 0	0 / 0	0 / 0
nitric oxide transport	4	4 / 1	0 / 0	0 / 0	4 / 0	0 / 0	0 / 0	0 / 0	0 / 0	0 / 0
skin morphogenesis	3	3 / 0	0 / 0	0 / 0	3 / 0	0 / 0	0 / 0	0 / 0	0 / 0	0 / 0
blood circulation	68	20 / 8	3 / 3	4 / 6	10 / 3	2 / 1	0 / 2	1 / 0	0 / 0	0 / 1
protein heterooligomerization	7	4 / 1	0 / 0	0 / 1	4 / 0	0 / 0	0 / 0	0 / 0	0 / 0	0 / 0
odontogenesis	16	6 / 2	0 / 1	0 / 1	6 / 1	0 / 0	0 / 0	0 / 0	0 / 0	0 / 0
erythrocyte maturation	8	4 / 1	1 / 0	0 / 1	4 / 0	0 / 0	0 / 0	0 / 0	0 / 0	0 / 0
cell junction assembly	6	4 / 1	0 / 0	0 / 1	3 / 0	0 / 0	0 / 0	0 / 0	0 / 0	0 / 0
transforming growth factor beta receptor signaling pathway	37	9 / 4	0 / 2	4 / 3	8 / 1	0 / 0	1 / 1	1 / 0	0 / 0	0 / 0
regulation of blood pressure	37	11 / 4	1 / 2	0 / 3	8 / 1	1 / 0	0 / 1	0 / 0	0 / 0	0 / 0
positive regulation of nitric oxide biosynthetic process	11	4 / 1	2 / 1	2 / 1	4 / 0	1 / 0	1 / 0	0 / 0	0 / 0	0 / 0
positive regulation of muscle contraction	7	3 / 1	0 / 0	1 / 1	3 / 0	0 / 0	1 / 0	0 / 0	0 / 0	0 / 0
glucose catabolic process	51	16 / 6	1 / 2	4 / 4	9 / 2	0 / 1	1 / 1	0 / 0	0 / 0	0 / 1
collagen catabolic process	8	4 / 1	0 / 0	0 / 1	3 / 0	0 / 0	0 / 0	0 / 0	0 / 0	0 / 0
negative regulation of cellular protein metabolic process	45	13 / 5	2 / 2	7 / 4	8 / 2	0 / 0	1 / 1	0 / 0	0 / 0	0 / 0
negative regulation of cellular component organization	28	10 / 3	0 / 1	2 / 2	6 / 1	0 / 0	0 / 1	0 / 0	0 / 0	0 / 0
system development	765	124 / 86	20 / 33	52 / 66	51 / 28	6 / 8	20 / 20	9 / 5	0 / 2	5 / 8
nucleotide-excision repair, DNA gap filling	10	0 / 1	5 / 0	4 / 1	0 / 0	1 / 0	0 / 0	0 / 0	0 / 0	0 / 0
DNA replication initiation	24	0 / 3	8 / 1	3 / 2	0 / 1	1 / 0	0 / 1	0 / 0	0 / 0	0 / 0
somatic hypermutation of immunoglobulin genes	4	0 / 1	3 / 0	0 / 0	0 / 0	1 / 0	0 / 0	0 / 0	0 / 0	0 / 0
nuclear mRNA splicing, via spliceosome	98	4 / 11	18 / 4	11 / 8	2 / 4	3 / 1	1 / 3	1 / 1	1 / 0	1 / 1
DNA unwinding during replication	8	0 / 1	4 / 0	0 / 1	0 / 0	1 / 0	0 / 0	0 / 0	0 / 0	0 / 0
rRNA processing	62	1 / 7	11 / 3	4 / 5	0 / 2	2 / 1	1 / 2	0 / 0	1 / 0	0 / 1
response to unfolded protein	37	1 / 4	8 / 2	8 / 3	1 / 1	1 / 0	4 / 1	0 / 0	1 / 0	1 / 0
RNA transport	38	2 / 4	8 / 2	3 / 3	2 / 1	1 / 0	1 / 1	1 / 0	1 / 0	0 / 0
somatic recombination of immunoglobulin gene segments	7	1 / 1	3 / 0	0 / 1	0 / 0	1 / 0	0 / 0	0 / 0	0 / 0	0 / 0
base-excision repair	12	0 / 1	4 / 1	0 / 1	0 / 0	1 / 0	0 / 0	0 / 0	0 / 0	0 / 0
response to ionizing radiation	15	0 / 2	4 / 1	2 / 1	0 / 1	3 / 0	0 / 0	0 / 0	0 / 0	0 / 0
response to UV	19	0 / 2	5 / 1	1 / 2	0 / 1	3 / 0	1 / 1	0 / 0	0 / 0	0 / 0
DNA-dependent DNA replication	47	0 / 5	15 / 2	6 / 4	0 / 2	4 / 1	0 / 1	0 / 0	0 / 0	0 / 1
cell cycle	293	22 / 33	32 / 13	56 / 25	11 / 11	7 / 3	21 / 8	0 / 2	3 / 1	8 / 3
cell division	90	5 / 10	6 / 4	31 / 8	3 / 3	0 / 1	6 / 2	0 / 1	0 / 0	3 / 1
regulation of cyclin-dependent protein kinase activity	23	0 / 3	1 / 1	9 / 2	0 / 1	1 / 0	2 / 1	0 / 0	0 / 0	1 / 0
chromosome condensation	6	0 / 1	0 / 0	4 / 1	0 / 0	0 / 0	0 / 0	0 / 0	0 / 0	1 / 0
protein folding	105	11 / 12	11 / 5	23 / 9	3 / 4	3 / 1	7 / 3	0 / 1	1 / 0	2 / 1
meiosis	37	1 / 4	7 / 2	14 / 3	0 / 1	0 / 0	5 / 1	0 / 0	1 / 0	4 / 0
mitotic sister chromatid segregation	9	0 / 1	1 / 0	6 / 1	0 / 0	0 / 0	1 / 0	0 / 0	0 / 0	3 / 0
ciliary or flagellar motility	27	3 / 3	1 / 1	10 / 2	3 / 1	0 / 0	6 / 1	0 / 0	0 / 0	2 / 0
M phase	101	4 / 11	9 / 4	34 / 9	1 / 4	0 / 1	11 / 3	0 / 1	1 / 0	7 / 1
microtubule-based movement	52	2 / 6	2 / 2	20 / 5	1 / 2	0 / 1	9 / 1	0 / 0	0 / 0	4 / 1
spermatogenesis	86	12 / 10	6 / 4	19 / 7	4 / 3	2 / 1	14 / 2	0 / 1	1 / 0	8 / 1
sperm motility	13	4 / 2	0 / 1	4 / 1	2 / 1	0 / 0	4 / 0	1 / 0	0 / 0	1 / 0
fertilization	21	1 / 2	0 / 1	5 / 2	0 / 1	0 / 0	4 / 1	0 / 0	0 / 0	1 / 0
spermatid development	18	1 / 2	0 / 1	6 / 2	1 / 1	0 / 0	3 / 1	0 / 0	0 / 0	3 / 0

		Differentially expressed in the trout			Correlated with mouse			Testis Specific		
		Somatic	Gonia	Meiotic/ p-meiotic	Somatic	Gonia	Meiotic/ p-meiotic	Somatic	Gonia	Meiotic/ p-meiotic
**Molecular Function**	5222	571	231	454	180	54	135	32	11	57
sodium:potassium-exchanging ATPase activity	3	3 / 0	0 / 0	0 / 0	2 / 0	0 / 0	0 / 0	0 / 0	0 / 0	0 / 0
oxygen transporter activity	11	8 / 1	0 / 1	0 / 1	7 / 0	0 / 0	0 / 0	0 / 0	0 / 0	0 / 0
hemoglobin alpha binding	4	4 / 0	0 / 0	0 / 0	4 / 0	0 / 0	0 / 0	0 / 0	0 / 0	0 / 0
hemoglobin beta binding	4	4 / 0	0 / 0	0 / 0	4 / 0	0 / 0	0 / 0	0 / 0	0 / 0	0 / 0
oxygen binding	17	9 / 2	0 / 1	0 / 2	7 / 1	0 / 0	0 / 1	0 / 0	0 / 0	0 / 0
structural constituent of cytoskeleton	21	10 / 2	0 / 1	2 / 2	7 / 1	0 / 0	0 / 1	0 / 0	0 / 0	0 / 0
cytoskeletal protein binding	196	42 / 21	5 / 9	17 / 17	19 / 7	3 / 2	3 / 5	1 / 1	0 / 0	1 / 2
platelet-derived growth factor binding	5	3 / 1	0 / 0	0 / 0	3 / 0	0 / 0	0 / 0	0 / 0	0 / 0	0 / 0
magnesium chelatase activity	4	0 / 0	4 / 0	0 / 0	0 / 0	1 / 0	0 / 0	0 / 0	0 / 0	0 / 0
unfolded protein binding	75	7 / 8	16 / 3	13 / 7	2 / 3	3 / 1	3 / 2	0 / 1	1 / 0	1 / 1
MutLalpha complex binding	4	0 / 0	3 / 0	0 / 0	0 / 0	1 / 0	0 / 0	0 / 0	0 / 0	0 / 0
single-stranded DNA binding	22	1 / 2	7 / 1	4 / 2	0 / 1	2 / 0	1 / 1	0 / 0	0 / 0	1 / 0
ubiquitin activating enzyme activity	5	0 / 1	3 / 0	0 / 0	0 / 0	1 / 0	0 / 0	0 / 0	0 / 0	0 / 0
RNA binding	351	16 / 38	38 / 16	35 / 31	6 / 12	4 / 4	7 / 9	1 / 2	4 / 1	2 / 4
threonine-type endopeptidase activity	19	0 / 2	6 / 1	1 / 2	0 / 1	0 / 0	0 / 1	0 / 0	0 / 0	0 / 0
ribonucleoprotein binding	25	0 / 3	7 / 1	0 / 2	0 / 1	2 / 0	0 / 1	0 / 0	0 / 0	0 / 0
nucleotide binding	1135	104 / 124	83 / 50	130 / 99	34 / 39	21 / 12	40 / 29	5 / 7	3 / 2	19 / 12
enzyme inhibitor activity	163	22 / 18	20 / 7	12 / 14	5 / 6	4 / 2	3 / 4	1 / 1	0 / 0	1 / 2
ATP-dependent DNA helicase activity	7	0 / 1	3 / 0	1 / 1	0 / 0	1 / 0	0 / 0	0 / 0	0 / 0	0 / 0
3'-5' exonuclease activity	18	0 / 2	5 / 1	1 / 2	0 / 1	1 / 0	0 / 1	0 / 0	1 / 0	0 / 0
microtubule motor activity	36	2 / 4	1 / 2	13 / 3	1 / 1	0 / 0	9 / 1	0 / 0	0 / 0	3 / 0
adenylate kinase activity	7	0 / 1	0 / 0	3 / 1	0 / 0	0 / 0	3 / 0	0 / 0	0 / 0	0 / 0
nucleobase, nucleoside, nucleotide kinase activity	34	3 / 4	1 / 2	7 / 3	1 / 1	1 / 0	6 / 1	0 / 0	0 / 0	1 / 0
shikimate kinase activity	11	0 / 1	0 / 1	4 / 1	0 / 0	0 / 0	3 / 0	0 / 0	0 / 0	0 / 0
DNA-directed RNA polymerase activity	29	2 / 3	2 / 1	4 / 3	0 / 1	0 / 0	0 / 1	0 / 0	0 / 0	3 / 0

		Differentially expressed in the trout			Correlated with mouse			Testis Specific		
		Somatic	Gonia	Meiotic/ p-meiotic	Somatic	Gonia	Meiotic/ p-meiotic	Somatic	Gonia	Meiotic/ p-meiotic
**Cellular Component**	4855	532	217	413	181	51	119	30	12	48
MHC protein complex	10	7 / 1	0 / 0	0 / 1	1 / 0	0 / 0	0 / 0	0 / 0	0 / 0	0 / 0
extracellular region	483	87 / 53	19 / 22	23 / 41	28 / 18	4 / 5	5 / 12	8 / 3	0 / 1	3 / 5
integral to plasma membrane	450	82 / 49	3 / 20	21 / 38	27 / 17	1 / 5	4 / 11	10 / 3	0 / 1	2 / 4
sodium:potassium-exchanging ATPase complex	3	3 / 0	0 / 0	0 / 0	2 / 0	0 / 0	0 / 0	0 / 0	0 / 0	0 / 0
plasma membrane	920	152 / 101	14 / 41	50 / 78	59 / 34	7 / 10	14 / 23	12 / 6	0 / 2	5 / 9
hemoglobin complex	8	7 / 1	0 / 0	0 / 1	7 / 0	0 / 0	0 / 0	0 / 0	0 / 0	0 / 0
basolateral plasma membrane	61	21 / 7	1 / 3	2 / 5	11 / 2	0 / 1	0 / 2	1 / 0	0 / 0	0 / 1
cortical cytoskeleton	13	7 / 1	0 / 1	2 / 1	5 / 1	0 / 0	0 / 0	0 / 0	0 / 0	0 / 0
spectrin	3	3 / 0	0 / 0	1 / 0	3 / 0	0 / 0	0 / 0	0 / 0	0 / 0	0 / 0
collagen type I	3	3 / 0	0 / 0	0 / 0	3 / 0	0 / 0	0 / 0	0 / 0	0 / 0	0 / 0
filamentous actin	7	4 / 1	0 / 0	0 / 1	3 / 0	0 / 0	0 / 0	0 / 0	0 / 0	0 / 0
melanosome	53	14 / 6	6 / 2	3 / 5	9 / 2	2 / 1	1 / 1	1 / 0	0 / 0	0 / 1
external side of plasma membrane	20	7 / 2	0 / 1	1 / 2	5 / 1	0 / 0	0 / 1	1 / 0	0 / 0	0 / 0
contractile fiber part	47	13 / 5	1 / 2	5 / 4	8 / 2	0 / 1	1 / 1	0 / 0	0 / 0	0 / 0
cytoplasmic vesicle membrane	47	4 / 5	0 / 2	1 / 4	2 / 2	0 / 1	0 / 1	3 / 0	0 / 0	0 / 1
chaperonin-containing T-complex	5	1 / 1	4 / 0	0 / 0	0 / 0	2 / 0	0 / 0	0 / 0	0 / 0	0 / 0
DNA replication factor C complex	4	0 / 0	3 / 0	1 / 0	0 / 0	0 / 0	0 / 0	0 / 0	0 / 0	0 / 0
Cajal body	5	0 / 1	3 / 0	1 / 0	0 / 0	0 / 0	0 / 0	0 / 0	0 / 0	0 / 0
proteasome core complex	18	0 / 2	6 / 1	1 / 2	0 / 1	0 / 0	0 / 0	0 / 0	0 / 0	0 / 0
nucleoplasm	386	25 / 42	40 / 17	44 / 33	13 / 14	6 / 4	15 / 10	0 / 2	3 / 1	3 / 4
intracellular part	3870	421 / 424	206 / 173	341 / 329	157 / 144	49 / 41	96 / 95	23 / 24	11 / 10	36 / 38
preribosome	7	0 / 1	3 / 0	0 / 1	0 / 0	1 / 0	0 / 0	0 / 0	0 / 0	0 / 0
nuclear replication fork	9	0 / 1	4 / 0	3 / 1	0 / 0	0 / 0	0 / 0	0 / 0	1 / 0	0 / 0
condensed chromosome kinetochore	9	0 / 1	2 / 0	5 / 1	0 / 0	0 / 0	1 / 0	0 / 0	0 / 0	1 / 0
centriole	3	0 / 0	0 / 0	3 / 0	0 / 0	0 / 0	2 / 0	0 / 0	0 / 0	0 / 0
axonemal dynein complex	6	0 / 1	0 / 0	6 / 1	0 / 0	0 / 0	6 / 0	0 / 0	0 / 0	2 / 0
condensed chromosome	31	0 / 3	5 / 1	13 / 3	0 / 1	0 / 0	2 / 1	0 / 0	0 / 0	4 / 0
microtubule	94	7 / 10	3 / 4	36 / 8	2 / 4	1 / 1	19 / 2	1 / 1	1 / 0	7 / 1
cilium	34	2 / 4	0 / 2	16 / 3	1 / 1	0 / 0	13 / 1	0 / 0	0 / 0	5 / 0
flagellum	53	7 / 6	1 / 2	14 / 5	4 / 2	0 / 1	10 / 1	1 / 0	0 / 0	4 / 1
axoneme	8	0 / 1	0 / 0	8 / 1	0 / 0	0 / 0	8 / 0	0 / 0	0 / 0	3 / 0
microtubule associated complex	50	3 / 6	1 / 2	18 / 4	1 / 2	0 / 1	12 / 1	0 / 0	0 / 0	4 / 1
