# Supplementary figures and images for: Expression profiling of rainbow trout testis development identifies evolutionary conserved genes involved in spermatogenesis
Source: BMC Genomics. 2009 Nov 20;10:546. doi: 10.1186/1471-2164-10-546 (PMC2786911; doi:10.1186/1471-2164-10-546)

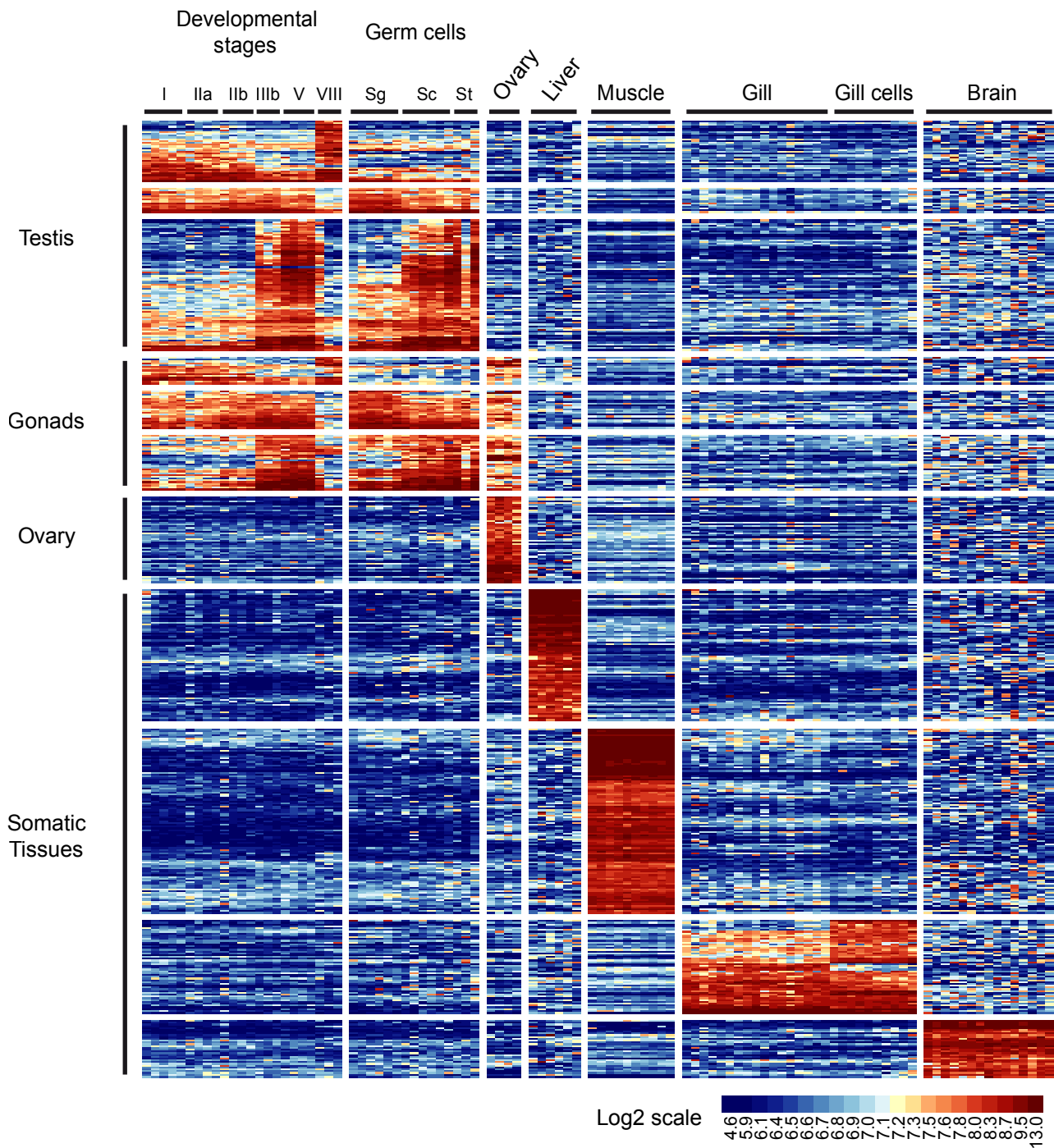

Supplement: Additional file 7 — Tissue-specific gene expression in trout. Tissue-specific genes were identified on the basis of their high expression (average signal intensity ≥3rd quartile) in at least one tissue (Testis, ovary, Liver, Muscle, Gill, or brain) and low or no expression (average signal intensity < median) in the 5 other tissues. Testis and Gonad specific genes are displayed according to 3 broad spermatogenesis expression clusters (i.e. somatic, spermatogonia and meiotic/post-meiotic). Log-2 transformed signal intensities are shown according to the scale bar. [file 1471-2164-10-546-S7.PDF]
