# Supplementary material for: Expression profiling of rainbow trout testis development identifies evolutionary conserved genes involved in spermatogenesis
Source: BMC Genomics. 2009 Nov 20;10:546. doi: 10.1186/1471-2164-10-546 (PMC2786911; doi:10.1186/1471-2164-10-546)
Supplement: Additional file 3 — Functional mining ("molecular function" and "cellular component") of trout spermatogenetic clusters. Over-represented "molecular function" and "cellular component" terms from the GeneOntology (GO) were identified in the 9 expression clusters shown in Figures 2 and 3 (A-I). Rectangles indicate the observed (left) and expected (right) numbers of genes bearing the corresponding GO term whereas the number of genes exhibiting this GO term on the entire microarray is given on the left. Only GO terms with a p-value ≤ 10-6 and for which at least 3 non-redundant genes belonged to the cluster were considered as statistically-enriched. To avoid redundancy between closely related terms an Ontology Specific Information Rate (OSIR) cut-off ≥0.95 was selected. Numbers in bold indicate a statistical enrichment for a given GO term according to the scale bar. [file 1471-2164-10-546-S3.PDF]

Depleted p-value Enriched

1 0.95 0.05 1e-5 0

		Somatic				Gonia		Meiotic/Post-Meiotic		
		"A"	"B"	"C"	"D"	"E"	"F"	"G"	"H"	"I"
**Molecular functions**	5222	261	118	131	81	90	144	101	257	98
sodium:potassium-exchanging ATPase activity	3	3 / 0	0 / 0	0 / 0	0 / 0	0 / 0	0 / 0	0 / 0	0 / 0	0 / 0
oxygen transporter activity	11	6 / 1	1 / 0	2 / 0	1 / 0	0 / 0	0 / 0	0 / 0	0 / 1	0 / 0
hemoglobin alpha binding	4	3 / 0	1 / 0	0 / 0	1 / 0	0 / 0	0 / 0	0 / 0	0 / 0	0 / 0
hemoglobin beta binding	4	3 / 0	1 / 0	0 / 0	1 / 0	0 / 0	0 / 0	0 / 0	0 / 0	0 / 0
oxygen binding	17	6 / 1	2 / 0	2 / 0	1 / 0	0 / 0	0 / 1	0 / 0	0 / 1	0 / 0
structural constituent of cytoskeleton	21	6 / 1	2 / 1	1 / 1	1 / 0	0 / 0	0 / 1	0 / 0	2 / 1	0 / 0
heme binding	86	14 / 4	4 / 2	3 / 2	2 / 1	2 / 2	1 / 2	0 / 2	4 / 4	1 / 2
platelet-derived growth factor binding	5	0 / 0	3 / 0	0 / 0	0 / 0	0 / 0	0 / 0	0 / 0	0 / 0	0 / 0
extracellular matrix structural constituent	24	2 / 1	5 / 1	1 / 1	0 / 0	0 / 0	1 / 1	0 / 1	0 / 1	1 / 1
G-protein-coupled receptor binding	16	0 / 1	0 / 0	5 / 0	0 / 0	0 / 0	0 / 0	0 / 0	0 / 1	0 / 0
anion exchanger activity	11	0 / 1	1 / 0	3 / 0	0 / 0	0 / 0	0 / 0	0 / 0	0 / 1	0 / 0
hydrolase activity, acting on carbon-nitrogen (but not peptide) bonds, in cyclic amidines	12	1 / 1	0 / 0	3 / 0	0 / 0	0 / 0	2 / 0	0 / 0	1 / 1	0 / 0
MHC class I receptor activity	5	1 / 0	0 / 0	0 / 0	3 / 0	0 / 0	0 / 0	0 / 0	0 / 0	0 / 0
glyceraldehyde-3-phosphate dehydrogenase (phosphorylating) activity	8	1 / 0	0 / 0	0 / 0	3 / 0	0 / 0	0 / 0	0 / 0	0 / 0	0 / 0
cysteine-type endopeptidase activity	49	5 / 2	1 / 1	1 / 1	5 / 1	2 / 1	1 / 1	0 / 1	2 / 2	1 / 1
enzyme inhibitor activity	163	10 / 8	6 / 4	4 / 4	4 / 3	12 / 3	8 / 5	2 / 3	5 / 8	5 / 3
ribonucleoprotein binding	25	0 / 1	0 / 1	0 / 1	0 / 0	4 / 0	3 / 1	0 / 1	0 / 1	0 / 1
alcohol dehydrogenase activity	26	4 / 1	1 / 1	1 / 1	2 / 0	4 / 0	2 / 1	0 / 1	0 / 1	1 / 1
magnesium chelatase activity	4	0 / 0	0 / 0	0 / 0	0 / 0	0 / 0	4 / 0	0 / 0	0 / 0	0 / 0
DNA-dependent ATPase activity	31	0 / 2	0 / 1	1 / 1	0 / 1	1 / 1	11 / 1	2 / 1	3 / 2	0 / 1
unfolded protein binding	75	3 / 4	2 / 2	2 / 2	0 / 1	2 / 1	15 / 2	5 / 2	6 / 4	2 / 1
ubiquitin activating enzyme activity	5	0 / 0	0 / 0	0 / 0	0 / 0	0 / 0	3 / 0	0 / 0	0 / 0	0 / 0
single-stranded DNA binding	22	0 / 1	0 / 1	0 / 1	1 / 0	1 / 0	6 / 1	1 / 0	3 / 1	0 / 0
DNA helicase activity	18	1 / 1	0 / 0	0 / 1	0 / 0	1 / 0	5 / 1	0 / 0	1 / 1	1 / 0
ATP-dependent helicase activity	43	2 / 2	0 / 1	0 / 1	0 / 1	1 / 1	8 / 1	0 / 1	3 / 2	0 / 1
single-stranded RNA binding	13	1 / 1	1 / 0	0 / 0	0 / 0	0 / 0	4 / 0	1 / 0	1 / 1	1 / 0
nucleotide binding	1135	52 / 57	17 / 26	21 / 29	17 / 18	24 / 20	60 / 31	33 / 22	77 / 56	21 / 21
ATP binding	782	31 / 39	12 / 18	15 / 20	13 / 12	17 / 14	43 / 22	23 / 15	55 / 39	15 / 15
hydrolase activity, acting on acid anhydrides, in phosphorus-containing anhydrides	474	26 / 24	4 / 11	9 / 12	5 / 7	9 / 8	30 / 13	14 / 9	39 / 23	8 / 9
ribonucleoside-diphosphate reductase activity	5	0 / 0	0 / 0	0 / 0	0 / 0	0 / 0	0 / 0	3 / 0	1 / 0	0 / 0
DNA topoisomerase (ATP-hydrolyzing) activity	16	0 / 1	0 / 0	0 / 0	0 / 0	0 / 0	0 / 0	3 / 0	1 / 1	1 / 0
microtubule motor activity	36	1 / 2	0 / 1	0 / 1	1 / 1	0 / 1	1 / 1	2 / 1	11 / 2	0 / 1
phosphotransferase activity, phosphate group as acceptor	24	2 / 1	0 / 1	0 / 1	0 / 0	1 / 0	0 / 1	0 / 1	7 / 1	0 / 1

		"A"	"B"	"C"	"D"	"E"	"F"	"G"	"H"	"I"
**Cellular components**	4855	251	113	115	72	78	142	98	226	91
hemoglobin complex	8	6 / 0	1 / 0	1 / 0	1 / 0	0 / 0	0 / 0	0 / 0	0 / 0	0 / 0
sodium:potassium-exchanging ATPase complex	3	3 / 0	0 / 0	0 / 0	0 / 0	0 / 0	0 / 0	0 / 0	0 / 0	0 / 0
membrane attack complex	178	23 / 9	5 / 4	7 / 4	1 / 3	0 / 3	0 / 5	1 / 4	7 / 8	1 / 3
basolateral plasma membrane	61	9 / 3	8 / 1	4 / 1	0 / 1	1 / 1	0 / 2	0 / 1	1 / 3	1 / 1
collagen type I	3	0 / 0	3 / 0	0 / 0	0 / 0	0 / 0	0 / 0	0 / 0	0 / 0	0 / 0
lateral plasma membrane	7	0 / 0	3 / 0	0 / 0	0 / 0	0 / 0	0 / 0	0 / 0	0 / 0	0 / 0
acrosome	8	1 / 0	3 / 0	0 / 0	0 / 0	0 / 0	0 / 0	0 / 0	0 / 0	0 / 0
cell-cell adherens junction	17	1 / 1	4 / 0	0 / 0	1 / 0	0 / 0	0 / 1	0 / 0	2 / 1	1 / 0
microvillus	12	1 / 1	3 / 0	0 / 0	0 / 0	0 / 0	0 / 0	0 / 0	0 / 1	0 / 0
soluble fraction	107	9 / 6	4 / 3	10 / 3	5 / 2	2 / 2	2 / 3	1 / 2	6 / 5	3 / 2
MHC class I protein complex	6	1 / 0	0 / 0	0 / 0	4 / 0	0 / 0	0 / 0	0 / 0	0 / 0	0 / 0
cytosol	581	31 / 30	13 / 14	17 / 14	23 / 9	17 / 9	28 / 17	18 / 12	33 / 27	8 / 11
I band	21	1 / 1	2 / 1	1 / 1	3 / 0	0 / 0	0 / 1	0 / 0	1 / 1	1 / 0
eukaryotic translation initiation factor 3 complex	9	0 / 1	0 / 0	1 / 0	0 / 0	3 / 0	0 / 0	0 / 0	1 / 0	0 / 0
proteasome core complex	18	0 / 1	0 / 0	0 / 0	0 / 0	3 / 0	3 / 1	1 / 0	0 / 1	0 / 0
chaperonin-containing T-complex	5	1 / 0	0 / 0	0 / 0	0 / 0	1 / 0	4 / 0	0 / 0	0 / 0	0 / 0
DNA replication factor C complex	4	0 / 0	0 / 0	0 / 0	0 / 0	0 / 0	3 / 0	1 / 0	0 / 0	0 / 0
nucleoplasm	386	11 / 20	5 / 9	4 / 9	5 / 6	4 / 6	36 / 11	16 / 8	22 / 18	6 / 7
intracellular part	3870	200/200	87/90	89/92	63/57	70/62	139/113	91/78	178/180	74/73
nuclear replisome	8	0 / 0	0 / 0	0 / 0	0 / 0	0 / 0	3 / 0	3 / 0	0 / 0	0 / 0
condensed chromosome, centromeric region	10	0 / 1	0 / 0	0 / 0	0 / 0	0 / 0	3 / 0	4 / 0	1 / 1	0 / 0
condensed chromosome kinetochore	9	0 / 1	0 / 0	0 / 0	0 / 0	0 / 0	2 / 0	4 / 0	1 / 0	0 / 0
microtubule	94	4 / 5	2 / 2	0 / 2	1 / 1	0 / 2	3 / 3	8 / 2	26 / 4	3 / 2
axonemal dynein complex	6	0 / 0	0 / 0	0 / 0	0 / 0	0 / 0	0 / 0	0 / 0	6 / 0	0 / 0
cilium	34	2 / 2	0 / 1	0 / 1	0 / 1	0 / 1	0 / 1	0 / 1	15 / 2	1 / 1
flagellum	53	2 / 3	1 / 1	1 / 1	3 / 1	1 / 1	0 / 2	0 / 1	13 / 3	1 / 1
centrosome	59	0 / 3	3 / 1	0 / 1	0 / 1	1 / 1	3 / 2	3 / 1	13 / 3	1 / 1
phosphorylase kinase complex	7	0 / 0	0 / 0	0 / 0	0 / 0	0 / 0	0 / 0	0 / 0	3 / 0	0 / 0
