# Supplementary material for: Expression profiling of rainbow trout testis development identifies evolutionary conserved genes involved in spermatogenesis
Source: BMC Genomics. 2009 Nov 20;10:546. doi: 10.1186/1471-2164-10-546 (PMC2786911; doi:10.1186/1471-2164-10-546)
Supplement: Additional file 8 — Trout clone annotation strategy. An ideogram presenting the annotation strategy for annotating a trout cDNA clone. BLAT alignments were performed on the fish species genomes available at the UCSC Genome Browser http://genome.ucsc.edu/. Annotation was performed using the Ensembl database http://www.ensembl.org/index.html version 52. [file 1471-2164-10-546-S8.PDF]

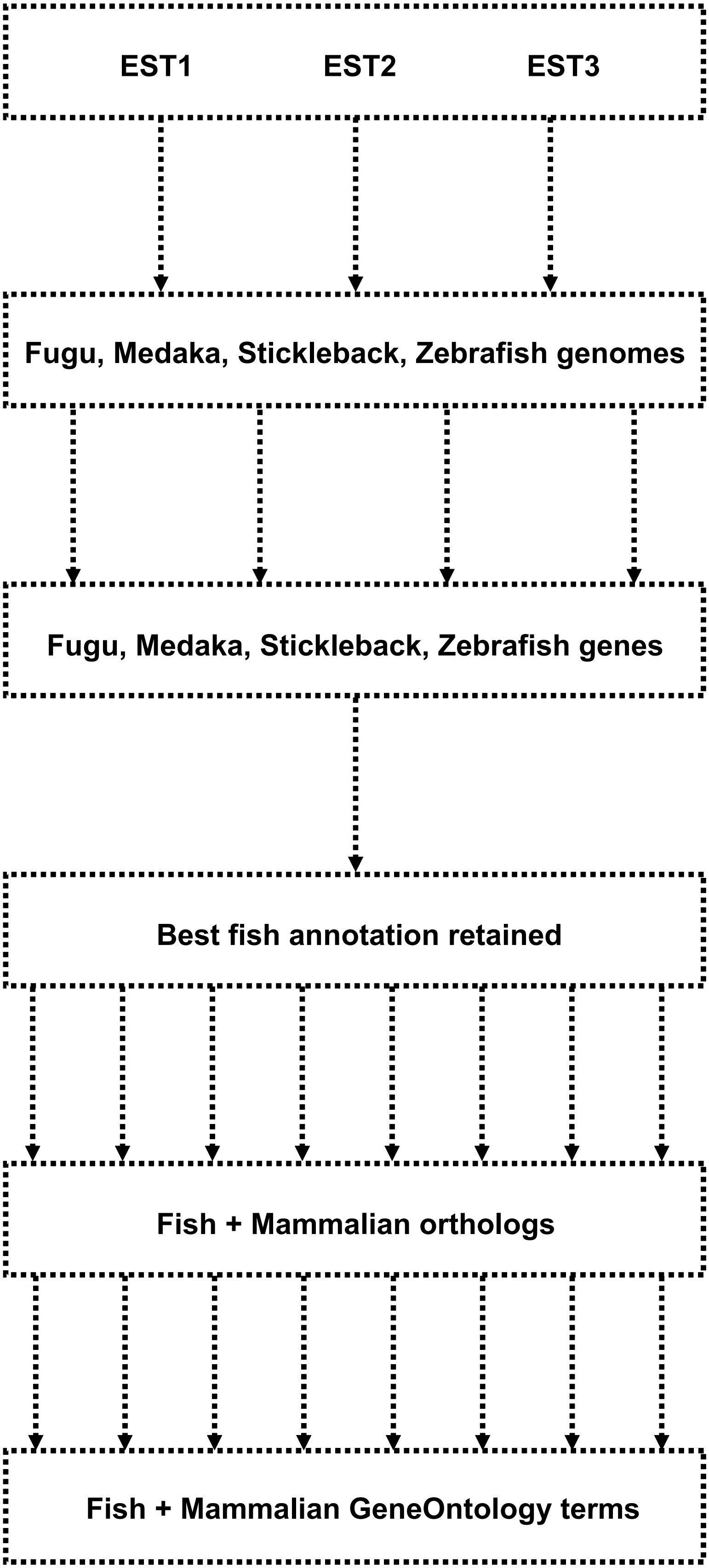

BLAT alignments
All EST are aligned on the 4 fish species genomes (RNA, DNA, translated RNA and translated DNA modes; masked repeats).

Initial cutoff: score> 30, homology> 60%

For each EST, the best localization(s) (best score + scores>0.95*[best scores]) on each genome are retained.

UCSC Genome Browser
Corresponding Ensembl gene IDs are extracted using the ensGene files.

Ensembl orthology:
The number (n) of orthologuous genes matched by each EST is counted. Each clone is then annotated with the EST exhibiting the highest "n". In the case when several EST have the same "n", the best score is then considered.

Ensembl Orthology and GeneOntology:
GeneOntology terms associated with fish as well as mouse, rat and human orthologuous genes are inferred to the trout clone.

Are finally not considered:
- ESTs with multiple localizations
- ESTs with score <50 and/or that mached a location >500 bp from the gene, if "n" <2
